# Hypervalent iodine(III)-mediated decarboxylative acetoxylation at tertiary and benzylic carbon centers

**DOI:** 10.3762/bjoc.14.92

**Published:** 2018-05-15

**Authors:** Kensuke Kiyokawa, Daichi Okumatsu, Satoshi Minakata

**Affiliations:** 1 Department of Applied Chemistry, Graduate School of Engineering, Osaka University, 2-1 Yamadaoka, Suita, Osaka 565-0871, Japan

**Keywords:** acetoxylation, carboxylic acids, decarboxylation, hypervalent iodine, iodine

## Abstract

The decarboxylative acetoxylation of carboxylic acids using a combination of PhI(OAc)_2_ and I_2_ in a CH_2_Cl_2_/AcOH mixed solvent is reported. The reaction was successfully applied to two types of carboxylic acids containing an α-quaternary and a benzylic carbon center under mild reaction conditions. The resulting acetates were readily converted into the corresponding alcohols by hydrolysis.

## Introduction

The decarboxylative functionalization of carboxylic acids and the derivatives thereof is an important transformation in organic synthesis. In recent years, increasing efforts have been devoted to the development of decarboxylative transformations [1–13], especially through radical decarboxylation processes, allowing an easy access to valuable compounds from readily available carboxylic acids. However, despite these advances, the oxidative decarboxylation coupled with C–O bond formation has received considerably less attention, even though it represents a promising strategy for the synthesis of valuable alcohol derivatives. One of the classical methods for the decarboxylative C–O bond formation of aliphatic carboxylic acids involves the use of stoichiometric amounts of heavy metal oxidants under high-temperature conditions [14–15]. Because these oxidants are typically highly toxic, their use has remained limited in organic synthesis. Barton et al. reported on the development of a practical method for the decarboxylative hydroxylation using thiohydroxamate esters as substrates [16]. Although the method was applicable to a broader range of substrates, the preparation of the activated ester is an intrinsic drawback to this procedure. While more convenient and practical methods for decarboxylative oxygenation, in which carboxylic acids are directly used as a substrate, have recently emerged, these methods have limited substrate scope [17–20].

**Scheme 1:**
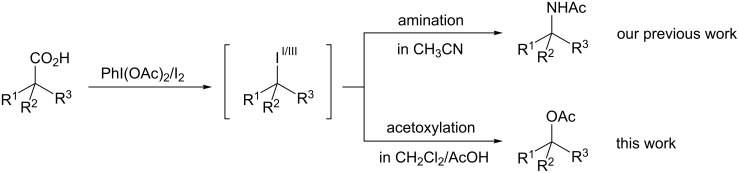
Decarboxylative functionalization using PhI(OAc)_2_/I_2_ system.

A seminal work on decarboxylative functionalization in which a combination of PhI(OAc)_2_ and molecular iodine (I_2_) are used was reported by Suárez et al. [21]. The method features mild reaction conditions, simple operation, and the use of readily available and environmentally friendly oxidants. However, despite the great potential of this approach with respect to a decarboxylative C–O bond-forming reaction, the oxidation system was only applied to reactions of uronic acids and α-amino acids [22–24], and further applications have not been explored. We recently reported on the decarboxylative Ritter-type amination of carboxylic acids containing an α-quaternary carbon center using a combination of PhI(OAc)_2_ and I_2_ to produce the corresponding α-tertiary amine derivatives (Scheme 1) [25]. Mechanistic investigations indicated that the reaction proceeds via the formation of an alkyl iodide and the corresponding iodine(III) species as key intermediates. In this context, we concluded that the use of such an oxidation system, combined with the judicious choice of solvent, would enable a decarboxylative C–O bond forming reaction, namely acetoxylation, via the oxidative displacement of an iodine atom of the in situ generated alkyl iodide by PhI(OAc)_2_ [26]. Herein, we report on the decarboxylative acetoxylation of carboxylic acids that contain an α-quaternary carbon center using PhI(OAc)_2_ and I_2_ in a CH_2_Cl_2_/AcOH mixed solvent (Scheme 1). In subsequent experiments, the method was also found to be applicable to the reaction of benzylic carboxylic acids. The acetates that were produced in the reaction were readily converted into the corresponding alcohols by hydrolysis.

## Results and Discussion

We started our investigation by examining the decarboxylative acetoxylation of 3-(4-bromophenyl)-2,2-dimethylpropanoic acid (**1a**) using PhI(OAc)_2_ and I_2_ as oxidants. When the reaction was conducted in AcOH, the corresponding acetate **2a** was obtained in low yield, and substantial amounts of the starting material were recovered, even though the use of AcOH as the solvent would be expected to promote the acetoxylation (Table 1, entry 1). Other solvents were then screened in attempts to improve the yield of the acetate **2a**. Halogenated solvents such as CH_2_Cl_2_, 1,2-dichloroethane, and chlorobenzene were found to be more effective in producing **2a** in moderate yields (Table 1, entries 2–4). The use of nitromethane also resulted in an improved yield of **2a** of 60% (Table 1, entry 5). Interestingly, screening of additional solvents revealed that a CH_2_Cl_2_/AcOH mixed solvent was suitable for this transformation, and a ratio of 1:1 (v/v) was found to be optimal, with **2a** being produced in 73% yield (Table 1, entries 6–8). When the reaction was performed at a higher concentration, the yield of product remained the same (Table 1, entry 9). Increasing the amount of oxidants used had only a slight effect on the product yield, with **2a** being produced in 77% yield, when 3 equiv of PhI(OAc)_2_ were used (Table 1, entries 10 and 11). The reaction efficiency was significantly decreased with a catalytic amount of I_2_, and no reaction was observed in the absence of I_2_ (Table 1, entries 12 and 13). The reaction did not proceed in the dark, and most of the starting material was recovered (Table 1, entry 14). This result is consistent with a reaction proceeding via a light-induced radical decarboxylation process [21,25].

**Table 1:** Effect of solvents and reaction parameters on the decarboxylative acetoxylation.^a^

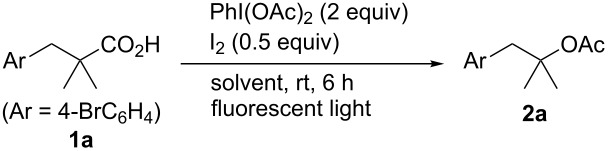		

Entry	Solvent	Yield (%)^b^

1	AcOH	10
2	CH_2_Cl_2_	48
3	1,2-dichloroethane	46
4	chlorobenzene	42
5	CH_3_NO_2_	60
6	CH_2_Cl_2_/AcOH (1:1)	73
7	CH_2_Cl_2_/AcOH (3:1)	57
8	CH_2_Cl_2_/AcOH (1:3)	65
9^c^	CH_2_Cl_2_/AcOH (1:1)	74
10^d^	CH_2_Cl_2_/AcOH (1:1)	77
11^e^	CH_2_Cl_2_/AcOH (1:1)	75
12^f^	CH_2_Cl_2_/AcOH (1:1)	8
13^g^	CH_2_Cl_2_/AcOH (1:1)	0
14^h^	CH_2_Cl_2_/AcOH (1:1)	<5

^a^Reactions were conducted on a 0.2 mmol scale at a 0.2 M concentration. Unless otherwise noted, reactions were performed on the benchtop with a fluorescent light on the ceiling. ^b^Determined by ^1^H NMR analysis of the crude product using 1,1,2,2-tetrachloroethane as an internal standard. ^c^The reaction was conducted at a 0.4 M concentration. ^d^PhI(OAc)_2_ (3 equiv) was used. ^e^I_2_ (1 equiv) was used. ^f^I_2_ (0.1 equiv) was used. ^g^The reaction was conducted without I_2_. ^h^The reaction was conducted in the dark.

**Scheme 2:**
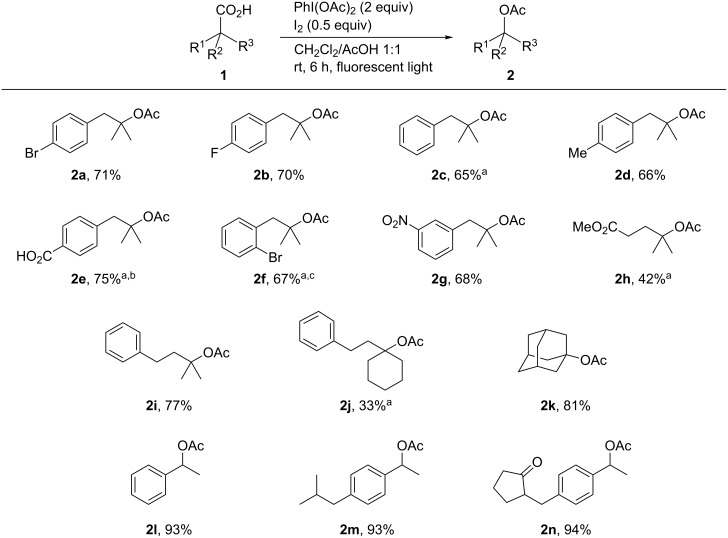
Substrate scope. Reactions were conducted on a 0.5 mmol scale at a 0.2 or 0.4 M concentration on the benchtop with a fluorescent light on the ceiling. Yields are for isolated products. ^a^PhI(OAc)_2_ (3 equiv) was used. ^b^I_2_ (1 equiv) was used. ^c^I_2_ (0.75 equiv) was used.

We next explored the scope of the decarboxylative acetoxylation reaction (Scheme 2). A variety of carboxylic acids containing α-quaternary carbon centers were efficiently converted into the corresponding acetates under mild reaction conditions [27]. Various functional groups, including bromo (**2a** and **2f**), fluoro (**2b**), carboxyl (**2e**), nitro (**2g**), and ester (**2h**) groups, were all well tolerated, providing the corresponding products in good yields. Notably, a carboxyl group on the phenyl ring was inert toward decarboxylation under the oxidation conditions used, allowing the acetate **2e** to be successfully synthesized. The acetoxylation of a cyclohexane framework was also achieved, but the yield of the product **2j** was somewhat lower than that of a non-cyclic **2i**. Using this protocol, 1-adamantanecarboxylic acid was smoothly transformed into the corresponding acetate **2k**. In addition to the reaction with respect to tertiary carbon centers, the present method was successfully applied to benzylic carboxylic acid derivatives. For example, commercially available arylpropanoic acids, which include ibuprofen (**1m**) and loxoprofen (**1n**), underwent decarboxylative acetoxylation in a highly efficient manner. It should be noted that, although benzylic C–H bonds are frequently incompatible with oxidative conditions, no products derived from benzylic C–H oxidation were observed in this reaction system.

**Scheme 3:**
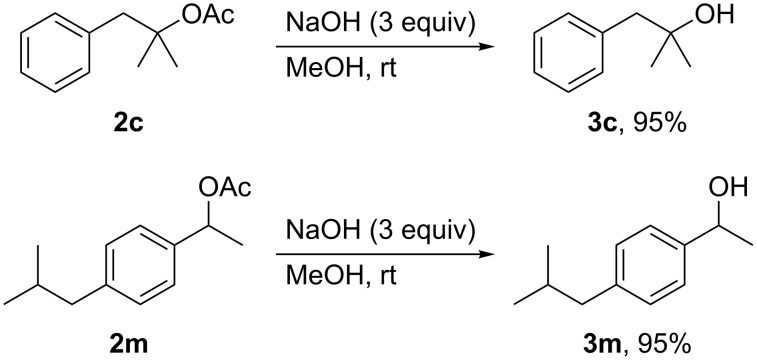
Hydrolysis of acetates.

Hydrolysis of the acetates **2c** and **2m** under basic conditions furnished the corresponding alcohols **3c** and **3m**, respectively, in nearly quantitative yields (Scheme 3). These results demonstrate that the present decarboxylative acetoxylation, followed by hydrolysis, offers an efficient and practical method for the synthesis of tertiary and benzylic alcohols.

**Scheme 4:**
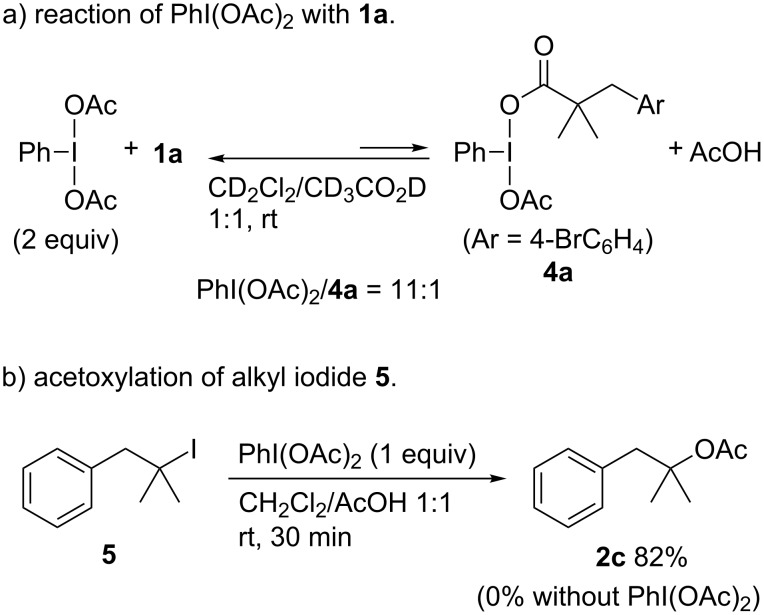
Mechanistic investigations.

The following experiments were performed in attempts to gain insights into the reaction pathway. Monitoring the reaction by ^1^H NMR spectroscopy showed for the reaction of a mixture of **1a** with two equimolar amounts of PhI(OAc)_2_ in a CD_2_Cl_2_/CD_3_CO_2_D (1:1) mixed solvent the formation of a mixture of PhI(OAc)_2_ and **4a** in a ratio of 11:1. This result indicates that ligand exchange between **1a** and PhI(OAc)_2_ was suppressed due to the presence of an excess amount of acetic acid (Scheme 4a, see Supporting Information File 1 for details). Based on our previous work, we propose that the reaction pathway involves the formation of an alkyl iodide and the corresponding tertiary alkyl-λ^3^-iodane species as intermediates [25]. To confirm the participation of these intermediates, the acetoxylation of a separately prepared sample of the tertiary alkyl iodide **5** was investigated. When **5** was treated with one equivalent of PhI(OAc)_2_ in a CH_2_Cl_2_/AcOH mixed solvent at room temperature, the acetoxylation proceeded efficiently to provide **2c** (Scheme 4b). On the contrary, in the absence of PhI(OAc)_2_, no products were formed, and the starting material was recovered. These results strongly support a reaction pathway involving the formation of an alkyl iodide, which is oxidized by PhI(OAc)_2_ to the corresponding hypervalent iodine(III) species that then undergoes acetoxylation.

**Scheme 5:**
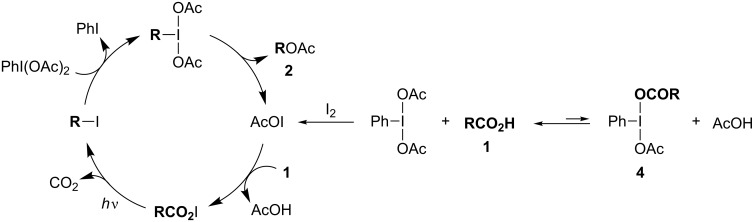
Proposed reaction pathway.

Based on the experimental results and our previous report [25], a proposed reaction pathway is depicted in Scheme 5. At the beginning of the reaction, PhI(OAc)_2_ predominantly exists rather than **4**, as confirmed by NMR analysis. Therefore, in the initial stage, PhI(OAc)_2_ preferentially undergoes decomposition with I_2_ to provide acetyl hypoiodite (AcOI), which participates in the generation of RCO_2_I by reacting with a carboxylic acid **1**. Meanwhile, RCO_2_I is directly generated along with AcOI when **4** reacts with I_2_. Subsequently, decarboxylative iodination of RCO_2_I under irradiation with visible light affords an alkyl iodide intermediate, which is then rapidly oxidized by the remaining PhI(OAc)_2_ to generate the corresponding alkyl-λ^3^-iodane (RI(OAc)_2_), which then undergoes the acetoxylation to afford the acetate **2** along with the regeneration of AcOI [28].

## Conclusion

In conclusion, the decarboxylative acetoxylation of carboxylic acids containing α-quaternary and benzylic carbon centers was achieved by using a combination of PhI(OAc)_2_ and I_2_ in a CH_2_Cl_2_/AcOH mixed solvent. The key to the success of the reaction was the choice of a suitable solvent that enables an oxidative substitution of an alkyl iodide intermediate by PhI(OAc)_2_. This operationally simple method under metal-free and mild reaction conditions can be used to produce a variety of acetates, which can be efficiently transformed into tertiary and benzylic alcohols.

## Supporting Information

File 1 Experimental procedures, characterization data, copies of the ^1^H, ^13^C, and ^19^F NMR spectra.
