# Supplementary material for: Hypervalent iodine(III)-mediated decarboxylative acetoxylation at tertiary and benzylic carbon centers
Source: Beilstein J Org Chem. 2018 May 15;14:1046–50. doi: 10.3762/bjoc.14.92 (PMC6009488; doi:10.3762/bjoc.14.92)

Supporting Information

for

Hypervalent iodine(III)-mediated decarboxylative acetoxylation at tertiary and benzylic carbon centers

Kensuke Kiyokawa*, Daichi Okumatsu and Satoshi Minakata*

Address: Department of Applied Chemistry, Graduate School of Engineering, Osaka University, 2-1 Yamadaoka, Suita, Osaka 565-0871, Japan

Email: Kensuke Kiyokawa - kiyokawa@chem.eng.osaka-u.ac.jp; Satoshi Minakata - minakata@chem.eng.osaka-u.ac.jp

* Corresponding author

Experimental procedures, characterization data, copies of the ^{1}H, ^{13}C, and ^{19}F NMR spectra

Table of Contents

1. General S2

2. Materials S2

3. ^{1}H NMR monitoring of the reaction mixture of $PhI(OAc)_2$ and **1a** S3

4. Acetoxylation of alkyl iodide **5** S3

5. Decarboxylative acetoxylation: typical procedure and product data S4

6. Hydrolysis of acetates S9

7. References S10

8. NMR spectra S11

1. General

New compounds were characterized by ^{1}H, ^{13}C, ^{19}F, IR, and HRMS. ^{1}H, ^{13}C, and ^{19}F NMR spectra were recorded on a JEOL JMTC-400/54/SS spectrometer (^{1}H NMR, 400 MHz; ^{13}C NMR, 100 MHz; ^{19}F NMR, 377 MHz). ^{1}H NMR chemical shifts were determined relative to Me_4Si (0.0 ppm) as an internal standard. ^{13}C NMR chemical shifts were determined relative to $CDCl_3$ (77.0 ppm). ^{19}F NMR chemical shifts were determined relative to C_6F_6 (−164.9 ppm) as an external standard. Infrared spectra were recorded on a SHIMADZU IRAffinity-1 FT-IR Spectrometer. High-resolution mass spectra were obtained on a JMS-700 mass spectrometer. Melting points were determined on a Stanford Research Systems MPA100 OptiMelt Automated Melting Point System. All reactions were carried out under nitrogen. Otherwise noted, reactions were performed on the benchtop with a fluorescent light on the ceiling. Products were purified by chromatography on silica gel BW-300 (Fuji Silysia Chemical Ltd.). Analytical thin-layer chromatography (TLC) was performed on pre-coated silica gel glass plates (Merck silica gel 60 F_{254} and Fuji Silysia Chromatorex NH, 0.25 mm thickness). Compounds were visualized with UV lamp or treatment with an ethanolic solution of phosphomolybdic acid followed by heating.

2. Materials

Starting materials **1a–j** were prepared according to literature procedures.[1] Alkyl iodide **5** was prepared according to a literature procedure.[1] All other starting materials, solvents, and reagents were purchased and used as obtained.

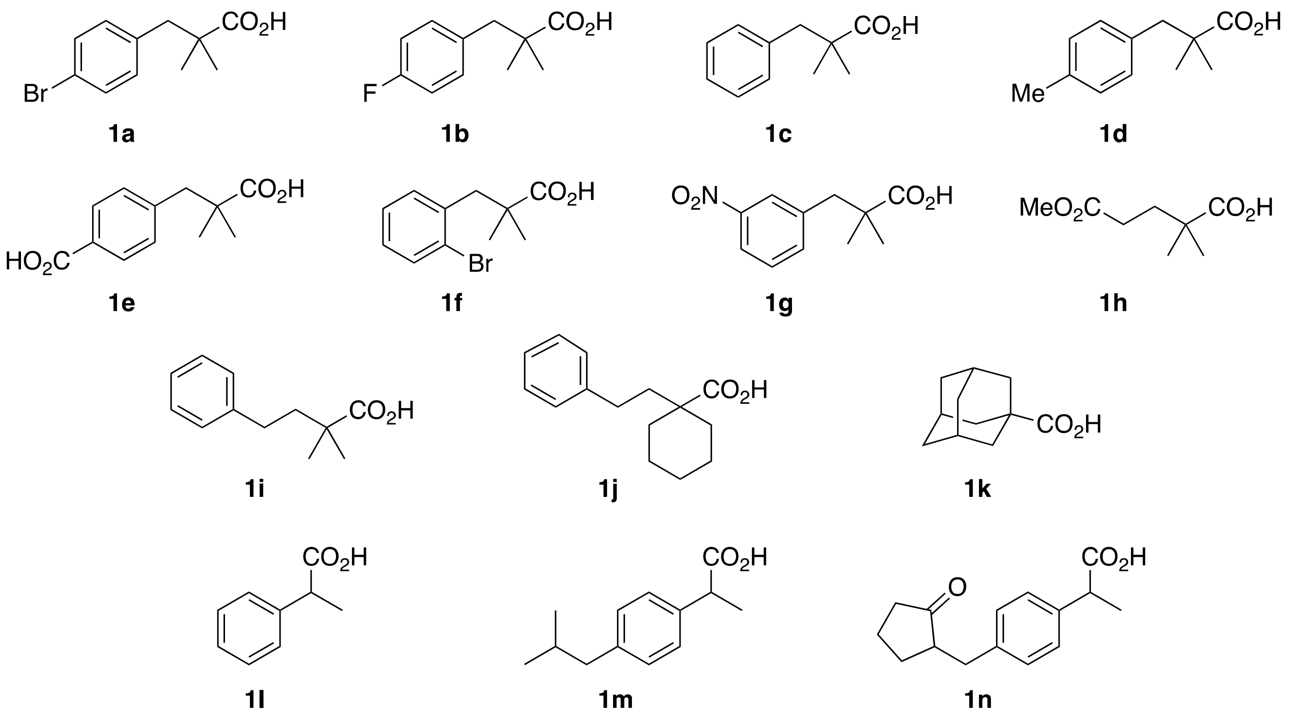

Figure S1: List of substrates.

3. ^{1}H NMR monitoring of the reaction mixture of PhI(OAc)$_2$ and 1a

The mixture of PhI(OAc)$_2$ (64.4 mg, 0.20 mmol) and 3-(4-bromophenyl)-2,2-dimethylpropanoic acid (**1a**, 25.8 mg, 0.10 mmol) in CD_2Cl_2/CD_3CO_2D (v/v = 1:1, 0.50 mL) was prepared. After mixing for 5 min at room temperature, the mixture was transferred into an NMR tube. The resulting ^{1}H NMR spectra are shown in Figure S2. The reaction provided a mixture of PhI(OAc)$_2$ and **4a** in a ratio of 11:1.

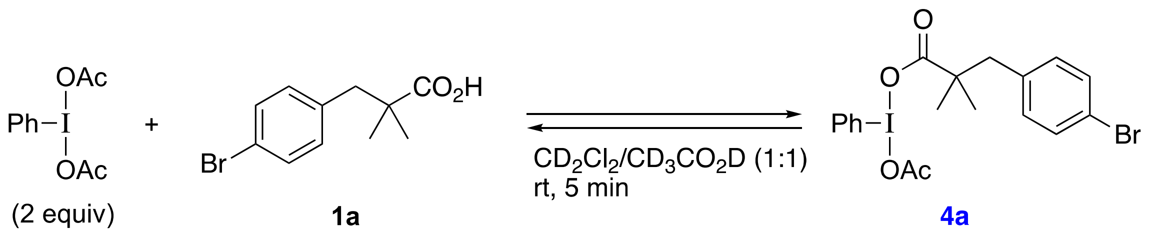

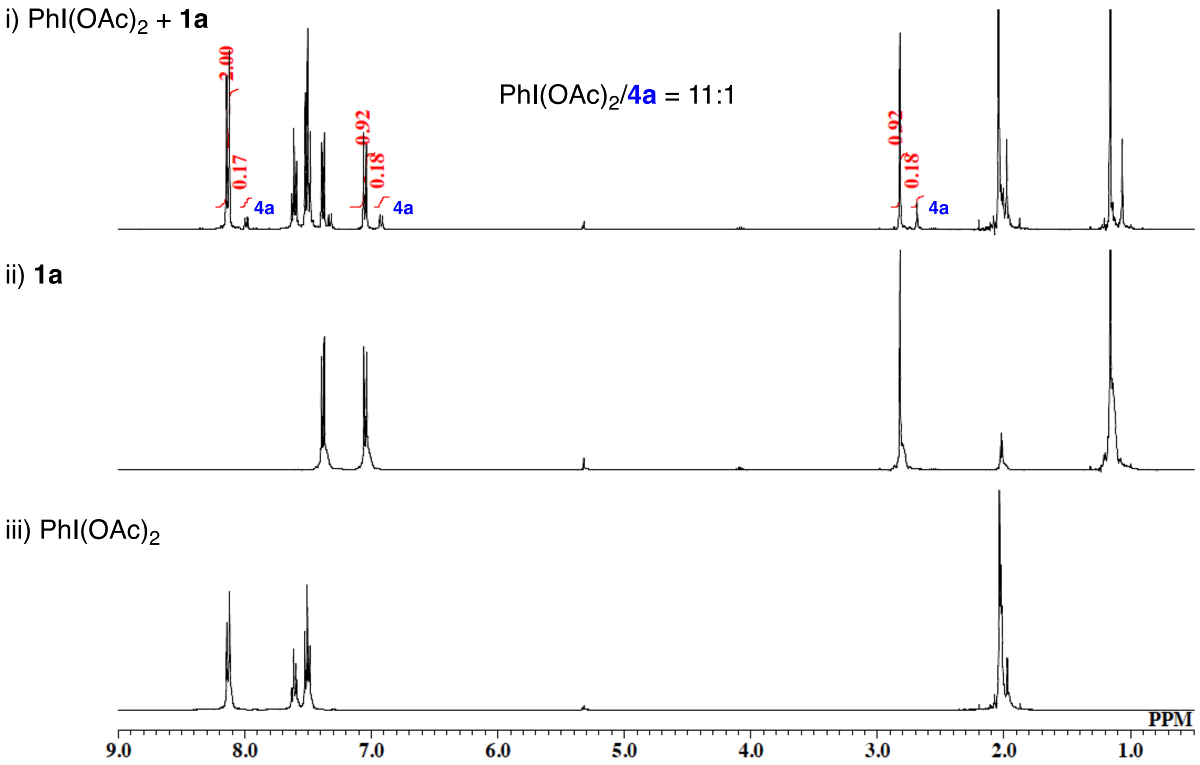

Figure S2: ^{1}H NMR spectra in CD_2Cl_2/CD_3CO_2D (v/v = 1:1). i) A mixture of **1a** and 2 equivalents of PhI(OAc)$_2$. ii) **1a**. iii) PhI(OAc)$_2$.

4. Acetoxylation of alkyl iodide 5

A heat-gun-dried two-necked reaction flask containing a magnetic stirring bar was charged with (2-iodo-2-methylpropyl)benzene (**5**) (53.9 mg, 0.21 mmol), PhI(OAc)$_2$ (64.5 mg, 0.20 mmol), and CH_2Cl_2/AcOH (v/v = 1:1, 1 mL). The reaction mixture was stirred for 30 min at room temperature. The reaction was quenched by $Na_2S_2O_3$ aq. (1 M, 5 mL) and sat. $NaHCO_3$ aq. (5 mL). The mixture

was extracted with Et_2O (3 x 10 mL)and the collected organic layers were dried over Na_2SO_4. The solution was concentrated under reduced pressure to give the crude product, which was analyzed by ^{1}H NMR spectroscopy using 1,1,2,2-tetrachloroethane as an internal standard.

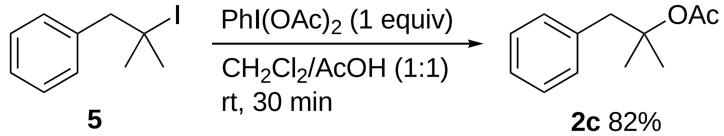

5. Decarboxylative acetoxylation: typical procedure and product data

Typical procedure: A heat-gun-dried two-necked reaction flask containing a magnetic stirring bar was charged with carboxylic acid **1** (0.5 mmol), $PhI(OAc)_2$ (1.00 or 1.50 mmol), and CH_2Cl_2/AcOH (v/v = 1:1, 1.25 or 2.5 mL). To the mixture, I_2 (0.25 or 0.38 mmol) was added, and the reaction mixture was stirred at room temperature for 6 h. The reaction was quenched by $Na_2S_2O_3$ aq. (1 M, 10 mL) and sat. $NaHCO_3$ aq. (10 mL). The mixture was extracted with Et_2O (3 × 20 mL). The collected organic layers were dried over Na_2SO_4. The solution was concentrated under reduced pressure to give the crude product, which was analyzed by ^{1}H NMR spectroscopy using 1,1,2,2-tetrachloroethane as an internal standard. Purification by flash column chromatography on silica gel (hexane/EtOAc) gave the pure product.

Product data

1-(4-Bromophenyl)-2-methylpropan-2-yl acetate (2a)

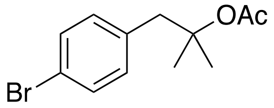

According to the typical procedure, the reaction using 3-(4-bromophenyl)-2,2-dimethylpropanoic acid (**1a**, 128.6 mg, 0.50 mmol), $PhI(OAc)_2$ (321.8 mg, 1.00 mmol), AcOH (0.63 mL), CH_2Cl_2 (0.63 mL), and I_2 (63.7 mg, 0.25 mmol) was conducted at room temperature for 6 h. Purification by flash column chromatography on silica gel (hexane/ethyl acetate = 95:5) gave the product as a colorless liquid (96.3 mg, 71% yield). ^{1}H NMR: (400 MHz, $CDCl_3$) δ 7.41 (d, J = 8.0 Hz, 2H), 7.06 (d, J = 8.0 Hz, 2H), 3.00 (s, 2H), 1.97 (s, 3H), 1.43 (s, 6H); ^{13}C NMR (100 MHz, $CDCl_3$) δ 170.5, 136.2, 132.1, 131.0, 120.4, 81.5, 45.7, 25.8, 22.5; IR: (ATR) 2976, 2930, 1732 cm^{-1}; HRMS: (FAB) calcd for ($C_{12}H_{14}BrO_2$) 269.0177 ($[M-H]^-$), found m/z 269.0170

1-(4-Fluorophenyl)-2-methylpropan-2-yl acetate (2b)

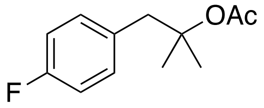

According to the typical procedure, the reaction using 3-(4-fluorophenyl)-2,2-dimethylpropanoic acid (**1b**, 98.3 mg, 0.50 mmol), $PhI(OAc)_2$ (320.2 mg, 1.00 mmol), AcOH (0.63 mL), CH_2Cl_2 (0.63 mL), and I_2 (63.3 mg, 0.25 mmol) was conducted at room temperature for 6 h. Purification by flash column chromatography on silica gel (hexane/ethyl acetate = 95:5) gave the product as a colorless liquid (74.1 mg, 70% yield). ^{1}H NMR: (400 MHz, $CDCl_3$) δ 7.18–7.11 (m, 2H), 7.02–6.94 (m, 2H), 3.02 (s, 2H), 1.97 (s, 3H), 1.43 (s, 6H); ^{13}C NMR (100 MHz, $CDCl_3$) δ 170.6, 161.7 (d, J_{CF} = 242.9 Hz), 132.9 (d, J_{CF} = 3.3 Hz), 131.8 (d, J_{CF} = 7.4 Hz), 114.7 (d, J_{CF} = 21.4 Hz), 81.8, 45.5, 25.9, 22.5; ^{19}F NMR (377 MHz, $CDCl_3$) δ –119.5; IR: (ATR) 2978, 2936, 1732 cm^{-1}; HRMS: (FAB) calcd for ($C_{12}H_{14}FO_2$) 209.0978 ([M−H]$^-$), found *m*/*z* 209.0975

2-Methyl-1-phenylpropan-2-yl acetate (2c)

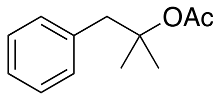

According to the typical procedure, the reaction using 2,2-dimethyl-3-phenylpropanoic acid (**1c**, 89.2 mg, 0.50 mmol), $PhI(OAc)_2$ (483.8 mg, 1.50 mmol), AcOH (1.25 mL), CH_2Cl_2 (1.25 mL), and I_2 (64.1 mg, 0.25 mmol) was conducted at room temperature for 6 h. Purification by flash column chromatography on silica gel (hexane/ethyl acetate = 95:5) gave the product as a colorless liquid (63.2 mg, 65% yield). ^{1}H NMR: (400 MHz, $CDCl_3$) δ 7.33–7.12 (m, 5H), 3.05 (s, 2H), 1.97 (s, 3H), 1.44 (s, 6H); ^{13}C NMR (100 MHz, $CDCl_3$) δ 170.6, 137.2, 130.5, 127.9, 126.4, 82.0, 46.3, 25.9, 22.5

The analytical data for this compound were in excellent agreement with the reported data.[2]

2-Methyl-1-(4-tolyl)propan-2-yl acetate (2d)

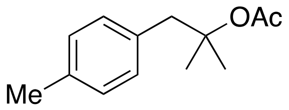

According to the typical procedure, the reaction using 2,2-dimethyl-3-(4-tolyl)propanoic acid (**1d**, 95.7 mg, 0.50 mmol), $PhI(OAc)_2$ (322.2 mg, 1.00 mmol), AcOH (0.63 mL), CH_2Cl_2 (0.63 mL), and I_2 (63.4 mg, 0.25 mmol) was conducted at room temperature for 6 h. Purification by flash column chromatography on silica gel (hexane/ethyl acetate = 95:5) gave the product as a colorless liquid (68.2 mg, 66% yield). ^{1}H NMR: (400 MHz, $CDCl_3$) δ 7.14–7.04 (m, 4H), 3.01 (s, 2H), 2.33 (s, 3H) 1.97 (s, 3H), 1.43 (s, 6H); ^{13}C NMR (100 MHz, $CDCl_3$) δ 170.7, 135.9, 134.2, 130.4, 128.7, 82.1, 45.9, 25.9, 22.6, 21.0; IR: (ATR) 2978, 2924, 1732 cm^{-1}; HRMS: (FAB) calcd for ($C_{13}H_{17}O_2$) 205.1229 ([M−H]$^-$), found *m*/*z* 205.1232

1-(4-Carboxyphenyl)-2-methylpropan-2-yl acetate (2e)

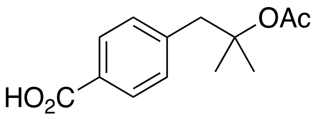

According to the typical procedure, the reaction using 3-(4-carboxyphenyl)-2,2-dimethylpropanoic acid (**1e**, 111.4 mg, 0.50 mmol), $PhI(OAc)_2$ (483.1 mg, 1.50 mmol), AcOH (1.25 mL), CH_2Cl_2 (1.25 mL), and I_2 (127.3 mg, 0.50 mmol) was conducted at room temperature for 6 h. Purification by flash column chromatography on silica gel (hexane/ethyl acetate = 90:10) gave the product as a white solid (88.8 mg, 75% yield). mp: 159.8–161.1 °C; ^{1}H NMR: (400 MHz, $CDCl_3$) δ 8.05 (d, *J* = 8.0 Hz, 2H), 7.30 (d, *J* = 8.0 Hz, 2H), 3.14 (s, 2H), 1.99 (s, 3H), 1.47 (s, 6H); ^{13}C NMR (100 MHz, $CDCl_3$) δ 171.6, 170.7, 143.8, 130.7, 129.9, 127.5, 81.7, 46.4, 26.1, 22.5; IR: (ATR) 2976, 2553, 1730, 1672 cm^{-1}; HRMS: (FAB) calcd for ($C_{13}H_{15}O_4$) 235.0970 ([M−H]$^-$), found *m*/*z* 235.0972

1-(2-Bromophenyl)-2-methylpropan-2-yl acetate (2f)

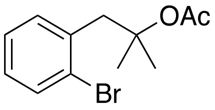

According to the typical procedure, the reaction using 3-(2-bromophenyl)-2,2-dimethylpropanoic acid (**1f**, 128.4 mg, 0.50 mmol), $PhI(OAc)_2$ (484.1 mg, 1.50 mmol), AcOH (1.25 mL), CH_2Cl_2 (1.25 mL), and I_2 (95.7 mg, 0.38 mmol) was conducted at room temperature for 6 h. Purification by flash column chromatography on silica gel (hexane/ethyl acetate = 95:5) gave the product as a colorless liquid (90.7 mg, 67% yield). ^{1}H NMR: (400 MHz, $CDCl_3$) δ 7.56 (dd, *J* = 8.0, 1.6 Hz, 1H), 7.33–7.21 (m, 2H), 7.09 (ddd, *J* = 8.0, 8.0, 2.0 Hz, 1H), 3.27 (s, 2H), 2.01 (s, 3H), 1.51 (s, 6H); ^{13}C NMR (100 MHz, $CDCl_3$) δ 170.5, 137.1, 133.0, 132.3, 128.1, 126.9, 126.1, 82.7, 45.2, 25.8, 22.6; IR: (ATR) 3062, 2980, 2935, 1732 cm^{-1}; HRMS: (FAB) calcd for ($C_{12}H_{14}BrO_2$) 269.0177 ([M−H]$^-$), found *m*/*z* 269.0172

2-Methyl-1-(3-nitrophenyl)propan-2-yl acetate (2g)

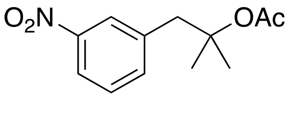

According to the typical procedure, the reaction using 2,2-dimethyl-3-(3-nitrophenyl)propanoic acid (**1g**, 111.2 mg, 0.50 mmol), $PhI(OAc)_2$ (320.2 mg, 0.99 mmol), AcOH (0.63 mL), CH_2Cl_2 (0.63 mL), and I_2 (63.6 mg, 0.25 mmol) was conducted at room temperature for 6 h. Purification by flash column chromatography on silica gel (hexane/ethyl acetate = 90:10) gave the product as a pale yellow solid (80.4 mg, 68% yield). mp: 76.9–78.1 °C; ^{1}H NMR: (400 MHz, $CDCl_3$) δ 8.16–8.07 (m, 2H), 7.57–7.45 (m, 2H), 3.15 (s, 2H), 2.00 (s, 3H), 1.47 (s, 6H); ^{13}C NMR (100 MHz, $CDCl_3$) δ 170.6, 148.0, 139.2, 136.7, 128.8, 125.2, 121.7, 81.2, 46.0, 25.9, 22.4; IR: (ATR) 2974, 2928, 1719 cm^{-1}; HRMS: (FAB) calcd for ($C_{12}H_{15}NO_4$) 237.1001 (M^-), found *m*/*z* 237.1006

4-Methoxycarbonyl-2-methylbutan-2-yl acetate (2h)

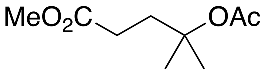

According to the typical procedure, the reaction using 2,2-dimethyl-4-methoxycarbonylbutanoic acid (**1h**, 82.8 mg, 0.48 mmol), $PhI(OAc)_2$ (484.7 mg, 1.50 mmol), AcOH (1.25 mL), CH_2Cl_2 (1.25 mL), and I_2 (63.3 mg, 0.25 mmol) was conducted at room temperature for 6 h. Purification by flash column chromatography on silica gel (hexane/ethyl acetate = 90:10) gave the product as a colorless liquid (37.6 mg, 42% yield). ^{1}H NMR: (400 MHz, $CDCl_3$) δ 3.68 (s, 3H), 2.43–2.35 (m, 2H), 2.11–2.03 (m, 2H), 1.97 (s, 3H), 1.46 (s, 6H); ^{13}C NMR (100 MHz, $CDCl_3$) δ 173.9, 170.3, 81.1, 51.7, 36.1, 29.0, 25.6, 22.3; IR: (ATR) 2980, 2953, 1732 cm^{-1}; HRMS: (FAB) calcd for ($C_9H_{15}O_4$) 187.0970 ($[M-H]^-$), found *m*/*z* 187.0966

2-Methyl-4-phenylbutan-2-yl acetate (2i)

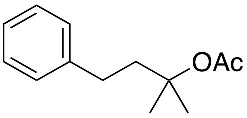

According to the typical procedure, the reaction using 2,2-dimethyl-4-phenylbutanoic acid (**1i**, 96.3 mg, 0.50 mmol), $PhI(OAc)_2$ (322.0 mg, 1.00 mmol), AcOH (0.63 mL), CH_2Cl_2 (0.63 mL), and I_2 (63.2 mg, 0.25 mmol) was conducted at room temperature for 6 h. Purification by flash column chromatography on silica gel (hexane/ethyl acetate = 95:5) gave the product as a colorless liquid (79.7 mg, 77% yield). ^{1}H NMR: (400 MHz, $CDCl_3$) δ 7.32–7.22 (m, 2H), 7.22–7.13 (m, 3H), 2.67–2.59 (m, 2H), 2.10–2.01 (m, 2H), 1.97 (s, 3H), 1.49 (s, 6H); ^{13}C NMR (100 MHz, $CDCl_3$) δ 170.4, 142.0, 128.3, 125.7, 81.8, 42.6, 30.3, 26.0, 22.3

The analytical data for this compound were in excellent agreement with the reported data.[3]

1-Phenethylcyclohexyl acetate (2j)

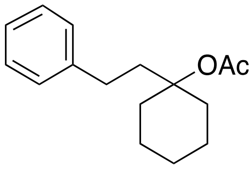

According to the typical procedure, the reaction using 1-phenethylcyclohexanecarboxylic acid (**1j**, 117.9 mg, 0.51 mmol), $PhI(OAc)_2$ (488.5 mg, 1.52 mmol), AcOH (1.25 mL), CH_2Cl_2 (1.25 mL), and I_2 (63.4 mg, 0.25 mmol) was conducted at room temperature for 6 h. Purification by flash column chromatography on silica gel (hexane/ethyl acetate = 95:5) gave the product as a colorless liquid (40.5 mg, 33% yield). ^{1}H NMR: (400 MHz, $CDCl_3$) δ 7.31–7.22 (m, 2H), 7.22–7.10 (m, 3H), 2.63–2.54 (m, 2H), 2.30–2.17 (m, 4H), 2.02 (s, 3H), 1.70–1.49 (m, 4H), 1.49–1.35 (m, 2H), 1.35–1.20 (m, 2H); ^{13}C NMR (100 MHz, $CDCl_3$) δ 170.4, 142.2, 128.4, 128.3, 125.7, 83.7, 39.4, 34.6, 29.6, 25.6, 22.2, 21.9; IR: (ATR) 2932, 2860, 1730 cm^{-1}; HRMS: (FAB) calcd for ($C_{16}H_{21}O_2$) 245.1452 ($[M-H]^-$), found *m*/*z* 245.1539

Adamantan-1-yl acetate (2k)

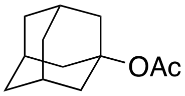

According to the typical procedure, the reaction using 1-adamantancarboxylic acid (**1k**, 90.6 mg, 0.50 mmol), $PhI(OAc)_2$ (322.2 mg, 1.00 mmol), AcOH (0.63 mL), CH_2Cl_2 (0.63 mL) and I_2 (63.8 mg, 0.25 mmol) was conducted at room temperature for 6 h. Purification by flash column chromatography on silica gel (hexane/ethyl acetate = 95:5) gave the product as a white solid (78.9 mg, 81% yield). ^{1}H NMR: (400 MHz, $CDCl_3$) δ 2.20–2.13 (m, 3H), 2.13–2.10 (m, 6H), 1.97 (s, 3H), 1.72–1.58 (m, 6H); ^{13}C NMR (100 MHz, $CDCl_3$) δ 170.3, 80.2, 41.3, 36.2, 30.7, 22.7
The analytical data for this compound were in excellent agreement with the reported data.[4]

1-Phenylethyl acetate (2l)

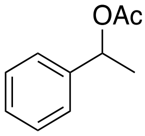

According to the typical procedure, the reaction using 2-phenylpropanoic acid (**1l**, 77.0 mg, 0.51 mmol), $PhI(OAc)_2$ (321.5 mg, 1.00 mmol), AcOH (1.25 mL), CH_2Cl_2 (1.25 mL), and I_2 (63.7 mg, 0.25 mmol) was conducted at room temperature for 6 h. Purification by flash column chromatography on silica gel (hexane/ethyl acetate = 95:5) gave the product as a colorless liquid (85.2 mg, 93% yield). ^{1}H NMR: (400 MHz, $CDCl_3$) δ 7.41–7.23 (m, 5H), 5.88 (q, *J* = 6.8 Hz, 1H), 2.06 (s, 3H), 1.53 (d, *J* = 6.8 Hz, 3H); ^{13}C NMR (100 MHz, $CDCl_3$) δ 170.3, 141.6, 128.5, 127.8, 126.1, 72.3, 22.2, 21.3
The analytical data for this compound were in excellent agreement with the reported data.[5]

1-(4-Isobutylphenyl)ethyl acetate (2m)

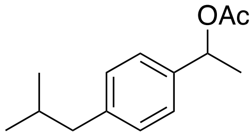

According to the typical procedure, the reaction using 2-(4-isobutylphenyl)propionic acid (**1m**, 103.0 mg, 0.50 mmol), $PhI(OAc)_2$ (322.1 mg, 1.00 mmol), AcOH (1.25 mL), CH_2Cl_2 (1.25 mL), and I_2 (63.7 mg, 0.25 mmol) was conducted at room temperature for 6 h. Purification by flash column chromatography on silica gel (hexane/ethyl acetate = 95:5) gave the product as a colorless liquid (102.6 mg, 93% yield). ^{1}H NMR: (400 MHz, $CDCl_3$) δ 7.26 (d, *J* = 8.0 Hz, 2H), 7.12 (d, *J* = 8.0 Hz, 2H), 5.87 (q, *J* = 6.4 Hz, 1H), 2.46 (d, *J* = 7.2 Hz, 2H), 2.06 (s, 3H), 1.92–1.78 (m, 1H), 1.53 (d, *J* = 6.4 Hz, 2H), 0.90 (d, *J* = 6.4 Hz, 6H); ^{13}C NMR (100 MHz, $CDCl_3$) δ 170.3, 141.4, 138.8, 129.1, 125.9, 72.2, 45.1, 30.1, 22.3, 22.0, 21.4; IR: (ATR) 2955, 2930, 2868, 1736, 1238 cm^{-1};HRMS: (FAB) calcd for ($C_{14}H_{20}O_2$) 220.1463 (M^+), found *m*/*z* 220.1464

1-[4-((2-Oxocyclopentyl)methyl)phenyl]ethyl acetate (2n)

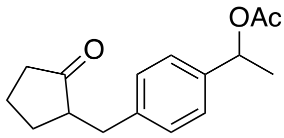

According to the typical procedure, the reaction using loxoprofen (**1n**, 125.1 mg, 0.50 mmol), $PhI(OAc)_2$ (323.0 mg, 1.00 mmol), AcOH (1.25 mL), CH_2Cl_2 (1.25 mL), and I_2 (64.0 mg, 0.25 mmol) was conducted at room temperature for 6 h. Purification by flash column chromatography on silica gel (hexane/ethyl acetate = 90:10) gave the product as a colorless liquid (124.2 mg, 94% yield). ^{1}H NMR: (400 MHz, $CDCl_3$) δ 7.27 (d, *J* = 8.0 Hz, 2H), 7.15 (d, *J* = 8.0 Hz, 2H), 5.86 (q, *J* = 6.4 Hz, 1H), 3.14 (dd, *J* = 14.0, 4.0 Hz, 1H), 2.52 (dd, *J* = 14.0, 9.6 Hz, 1H), 2.41–2.29 (m, 2H), 2.19–2.05 (m, 2H), 2.07 (s, 3H), 2.01–1.92 (m, 1H), 1.81–1.64 (m, 1H), 1.61–1.49 (m, 1H), 1.52 (d, *J* = 6.4 Hz, 3H); ^{13}C NMR (100 MHz, $CDCl_3$) δ 220.1, 170.3, 139.7, 139.5, 129.0, 126.2, 72.1, 51.0, 38.1, 35.2, 29.2, 22.1, 21.4, 20.5; IR: (ATR) 2963, 2934, 2874, 1732 cm^{-1}; HRMS: (FAB) calcd for ($C_{16}H_{19}O_3$) 259.1334 ([M−H]$^-$), found *m*/*z* 259.1328

6. Hydrolysis of acetates

2-Methyl-1-phenylpropan-2-ol (3c)

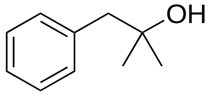

A two-necked reaction flask containing a magnetic stirring bar was charged with 1,1-dimethyl-2-phenylethyl acetate (**2c**, 95.0 mg, 0.49 mmol), NaOH (62.7 mg, 1.57 mmol), and MeOH (3 mL). The reaction mixture was stirred at room temperature for 24 h. The reaction was neutralized with HCl aq. (2 M), and the mixture was extracted with EtOAc (3 × 20 mL). The collected organic layers were dried over Na_2SO_4. The solution was concentrated under reduced pressure to give the crude product, which was analyzed by ^{1}H NMR spectroscopy using 1,1,2,2-tetrachloroethane as an internal standard. Purification by flash column chromatography on silica gel (hexane/ethyl acetate = 85:15) gave the product as a colorless liquid (70.5 mg, 95% yield). ^{1}H NMR: (400 MHz, $CDCl_3$) δ 7.46–7.17 (m, 5H), 2.77 (s, 2H), 1.23 (s, 6H); ^{13}C NMR (100 MHz, $CDCl_3$) δ 137.7, 130.4, 128.2, 126.5, 70.7, 49.7, 29.1

The analytical data for this compound were in excellent agreement with the reported data.[6]

1-(4-Isobutylphenyl)ethan-1-ol (3m)

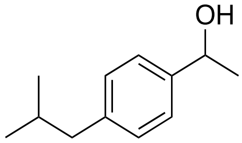

Two-necked reaction flask containing a magnetic stirring bar was charged with 1-(4-isobutylphenyl)ethyl acetate (**2m**, 116.3 mg, 0.53 mmol), NaOH (60.3 mg, 1.50 mmol), and MeOH (3 mL). The reaction mixture was stirred at room temperature for 24 h. The reaction was neutralized with HCl aq. (2 M), and the mixture was extracted with EtOAc (3 × 20 mL). The collected organic layers were dried over Na_2SO_4. The solution was concentrated under reduced pressure to give the crude product, which was analyzed by ^{1}H NMR spectroscopy using 1,1,2,2-tetrachloroethane as an internal standard. Purification by flash column chromatography on silica gel (hexane/ethyl acetate = 85:15) gave the product as a colorless liquid (89.4 mg, 95% yield). ^{1}H NMR: (400 MHz, $CDCl_3$) δ 7.28 (d, *J* = 8.0 Hz, 2H), 7.13 (d, *J* = 8.0 Hz, 2H), 4.91–4.85 (m, 1H), 2.46 (d, *J* = 6.8 Hz, 2H), 1.90–1.80 (m, 1H), 1.77 (brs, 1H), 1.49 (d, *J* = 6.4 Hz, 3H), 0.90 (d, *J* = 6.8 Hz, 6H); ^{13}C NMR (100 MHz, $CDCl_3$) δ 143.0, 141.0, 129.2, 125.2, 70.3, 45.1, 30.2, 25.0, 22.4

The analytical data for this compound were in excellent agreement with the reported data.[7]

7. References

(1) Kiyokawa, K.; Watanabe, T.; Fra, L.; Kojima, T.; Minakata, S. *J. Org. Chem.* **2017**, *82*, 11711.

(2) Chakraborti, A. K.; Shivani *J. Org. Chem.* **2006**, *71*, 5785.

(3) Nishimoto, Y.; Okita, A.; Yasuda, M.; Baba, A. *Org. Lett.* **2012**, *14*, 1846.

(4) Liu, Z.; Ma, Q.; Liu, Y.; Wang, Q. *Org. Lett.* **2014**, *16*, 236.

(5) Yu, W.; Zhou, M.; Wang, T.; He, Z.; Shi, B.; Xu, Y.; Huang, K. *Org. Lett.* **2017**, *19*, 5776.

(6) Liu, Z.; Zhang, Y.; Cai, Z.; Sun, H.; Cheng, X. *Adv. Synth. Catal.* **2015**, *357*. 589.

(7) Song, H.-T.; Ding, W.; Zhou, Q.-Q.; Liu, J.; Lu, L.-Q.; Xiao, W.-J. *J. Org. Chem.* **2016**, *81*, 7250.

8. NMR spectra

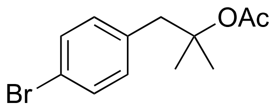

2a

^{1}H NMR: (400 MHz, $CDCl_3$)

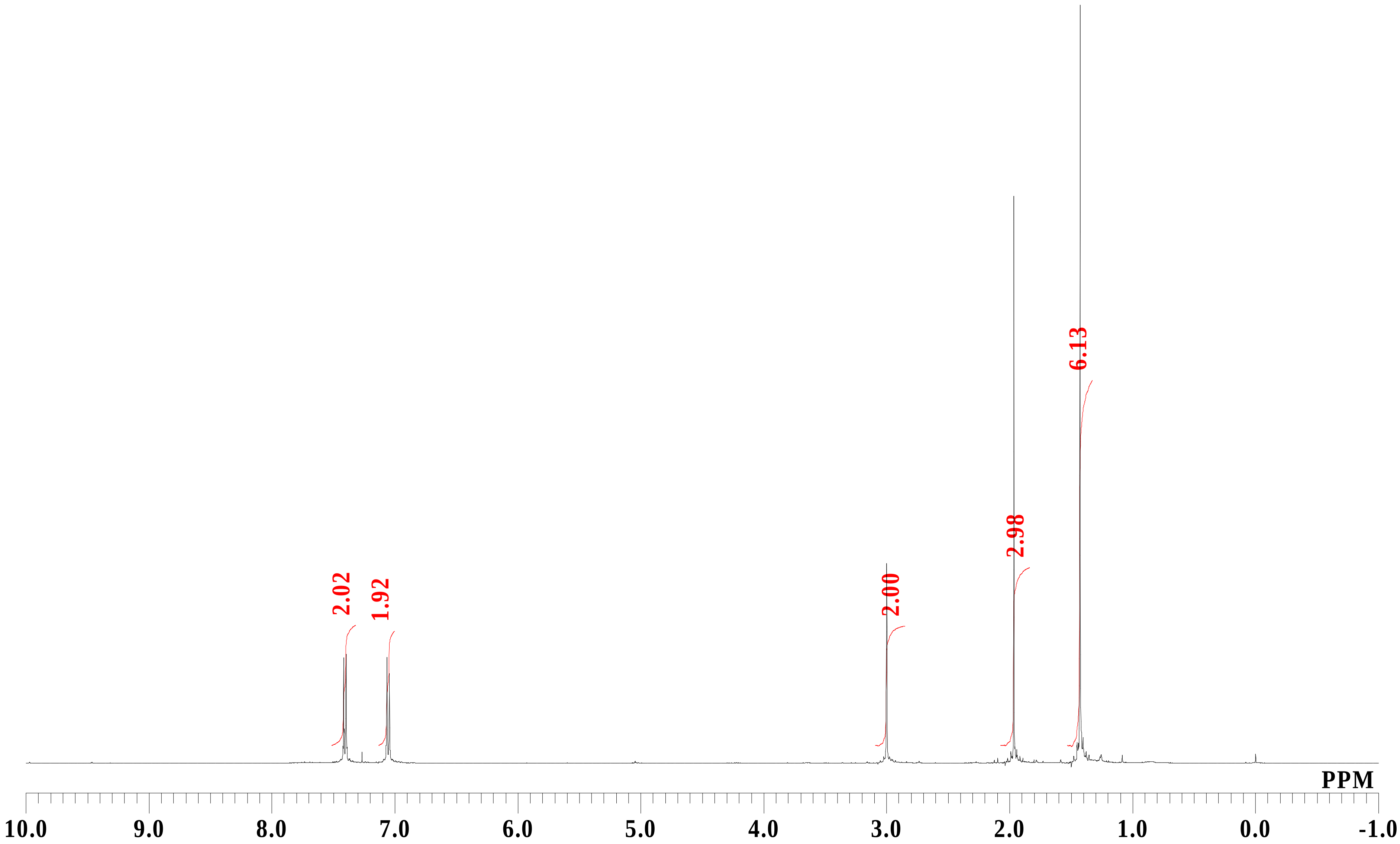

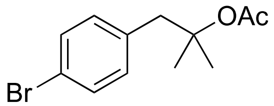

2a

^{13}C NMR: (100 MHz, $CDCl_3$)

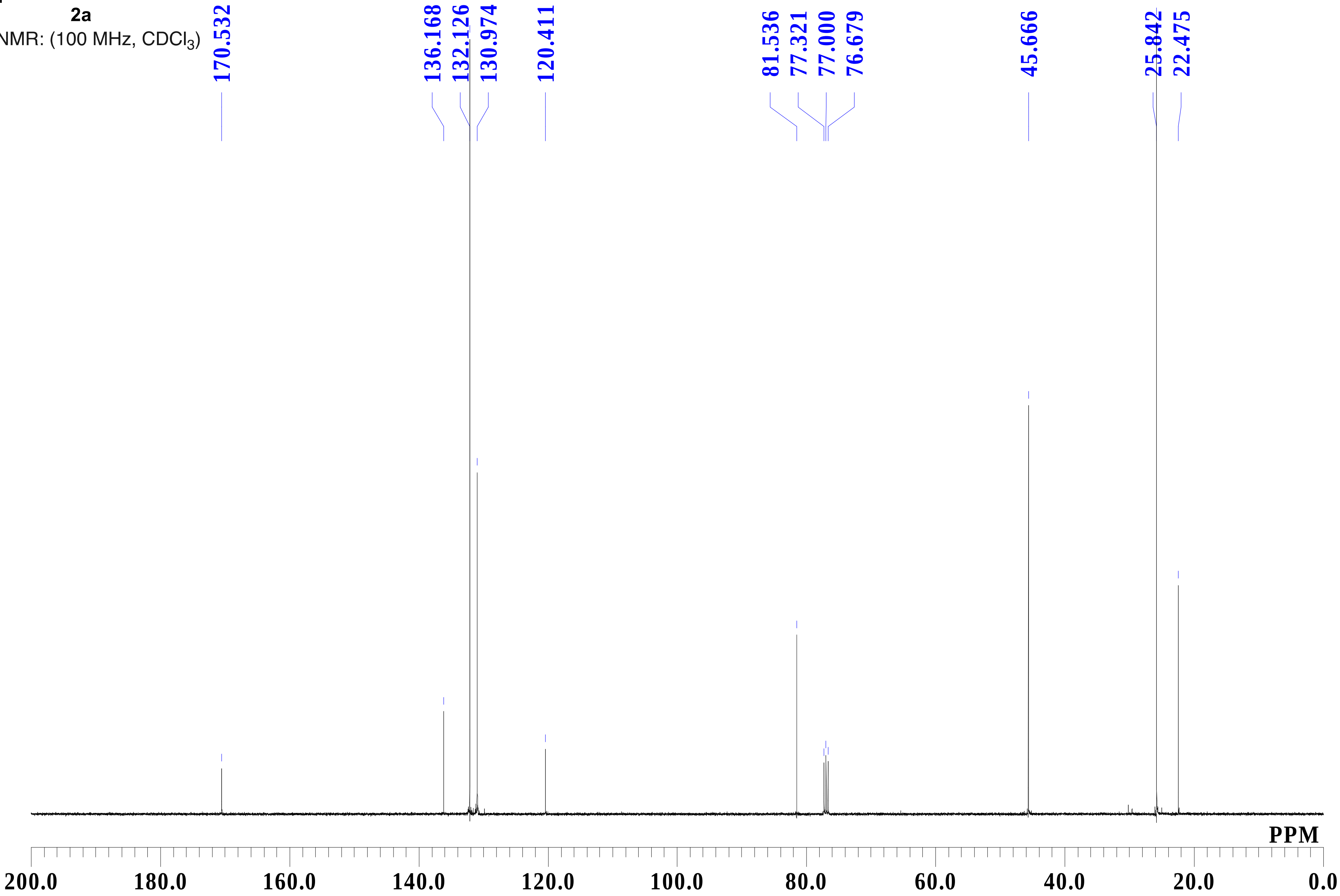

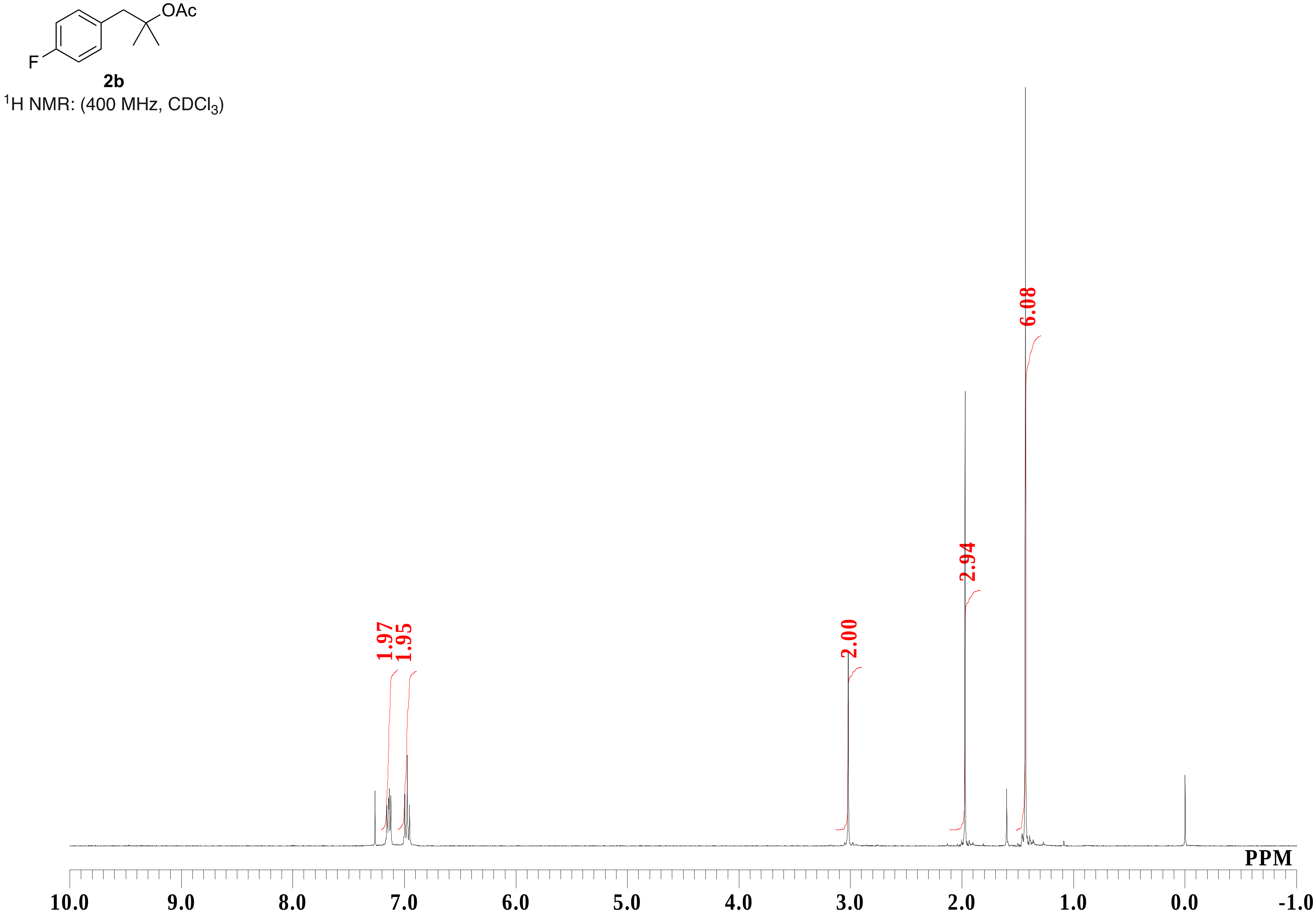
OAc
F
2b
1H NMR: (400 MHz, CDCl3)
1.97
1.95
2.00
2.94
6.08
PPM
10.0
9.0
8.0
7.0
6.0
5.0
4.0
3.0
2.0
1.0
0.0
-1.0

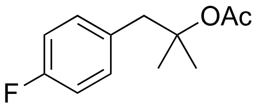

2b

^{13}C NMR: (100 MHz, $CDCl_3$)

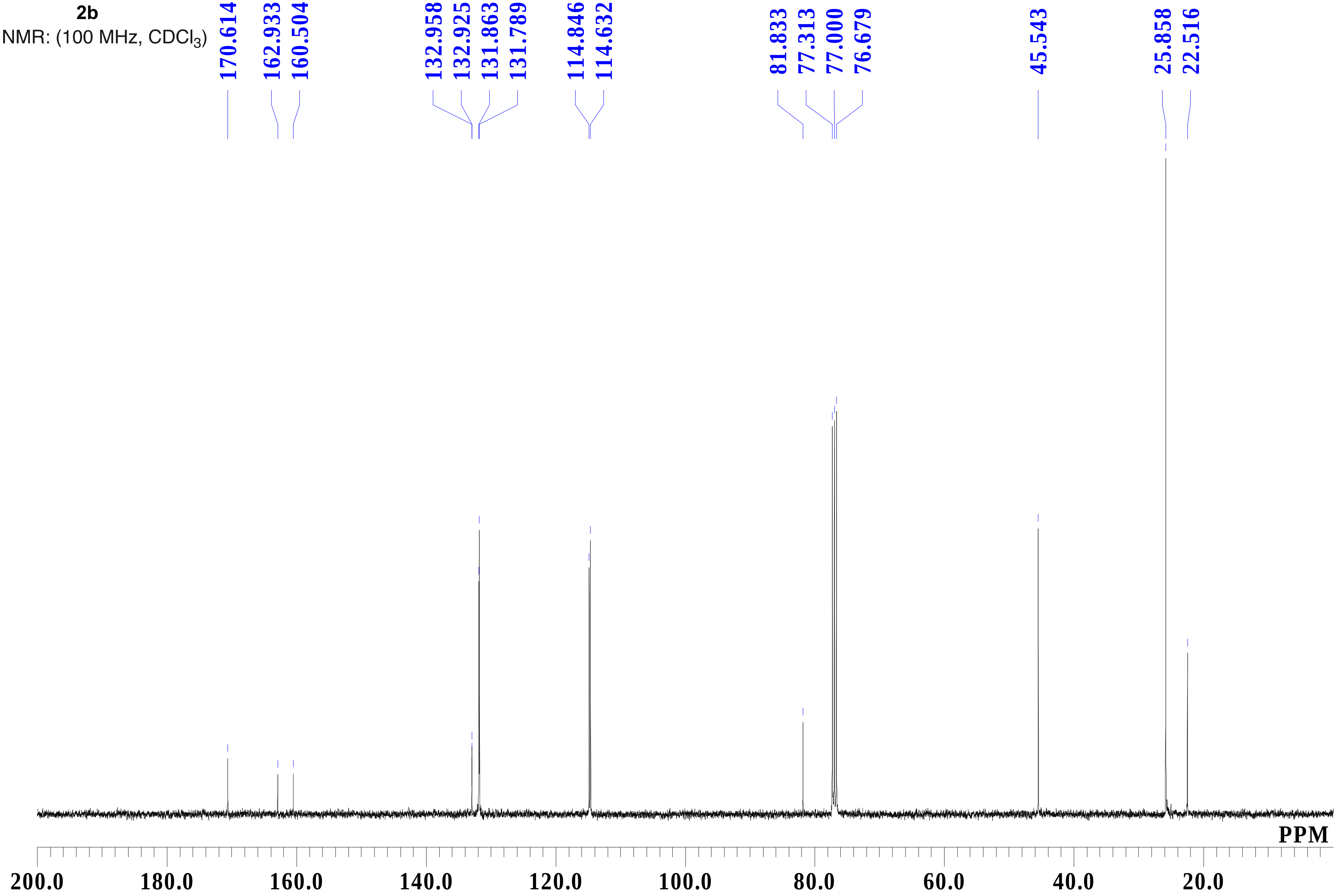

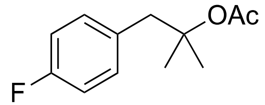

2b

^{19}F NMR: (377 MHz, $CDCl_3$)

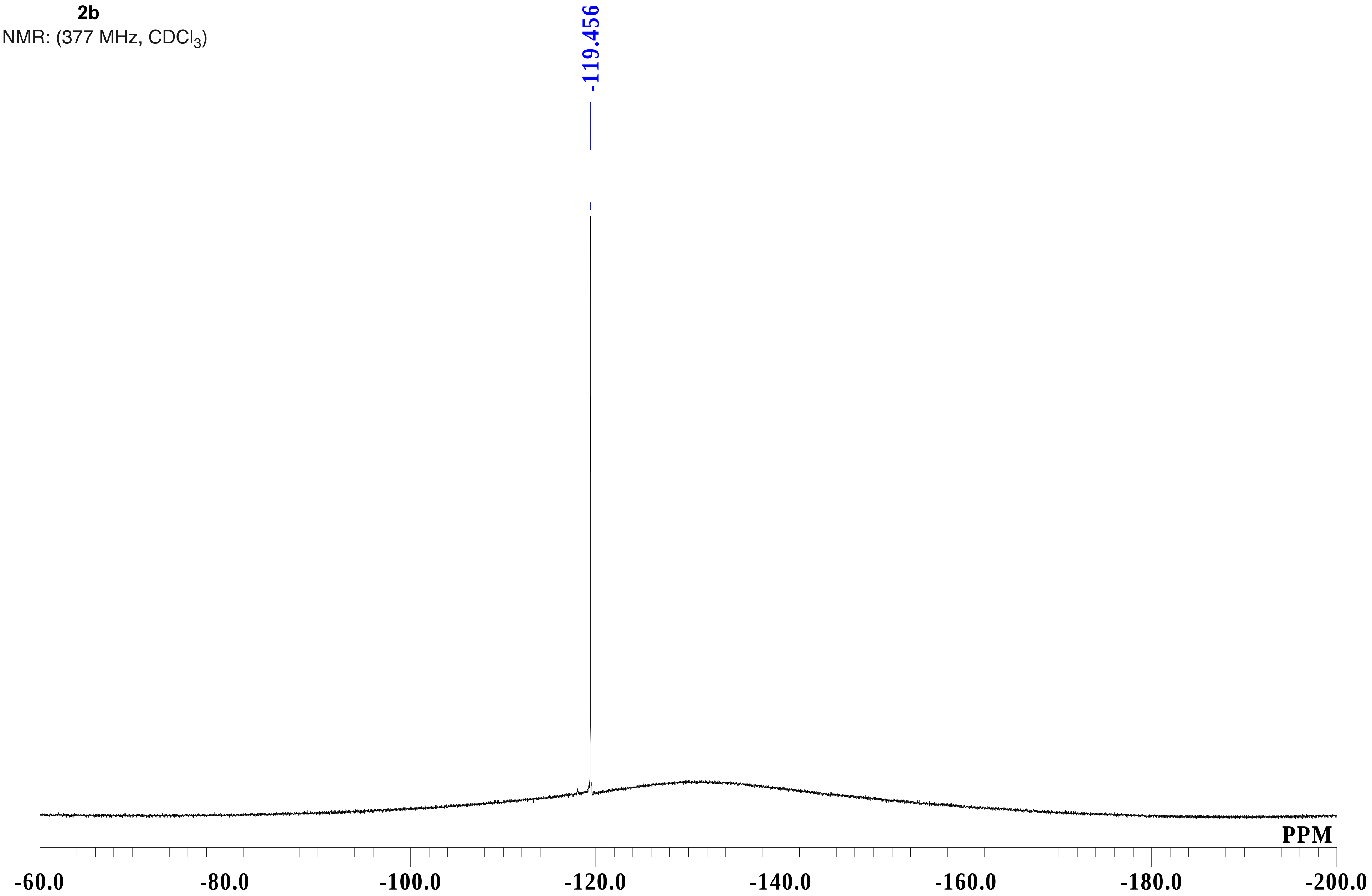

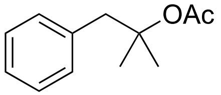

2c

^{1}H NMR: (400 MHz, $CDCl_3$)

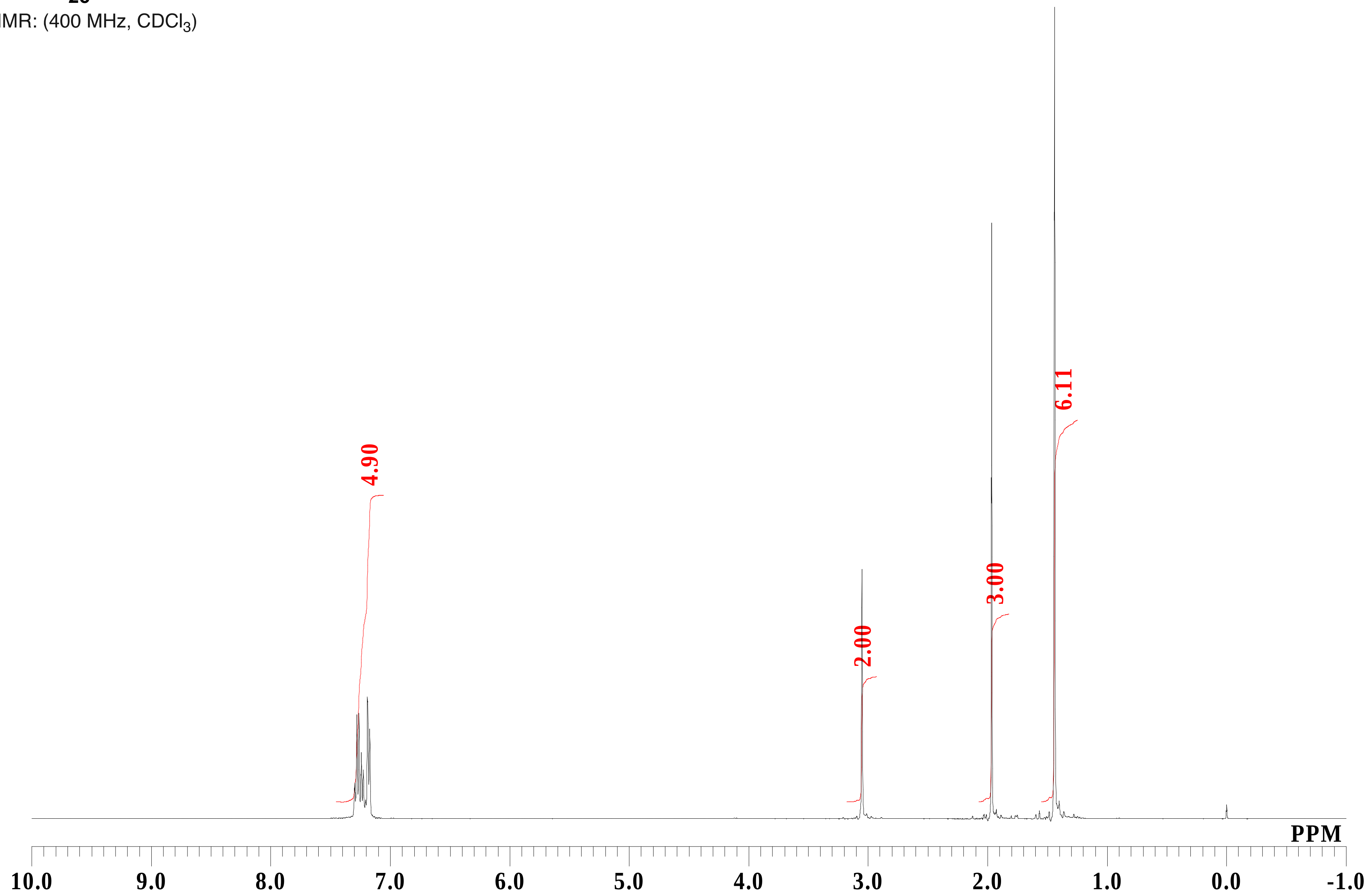

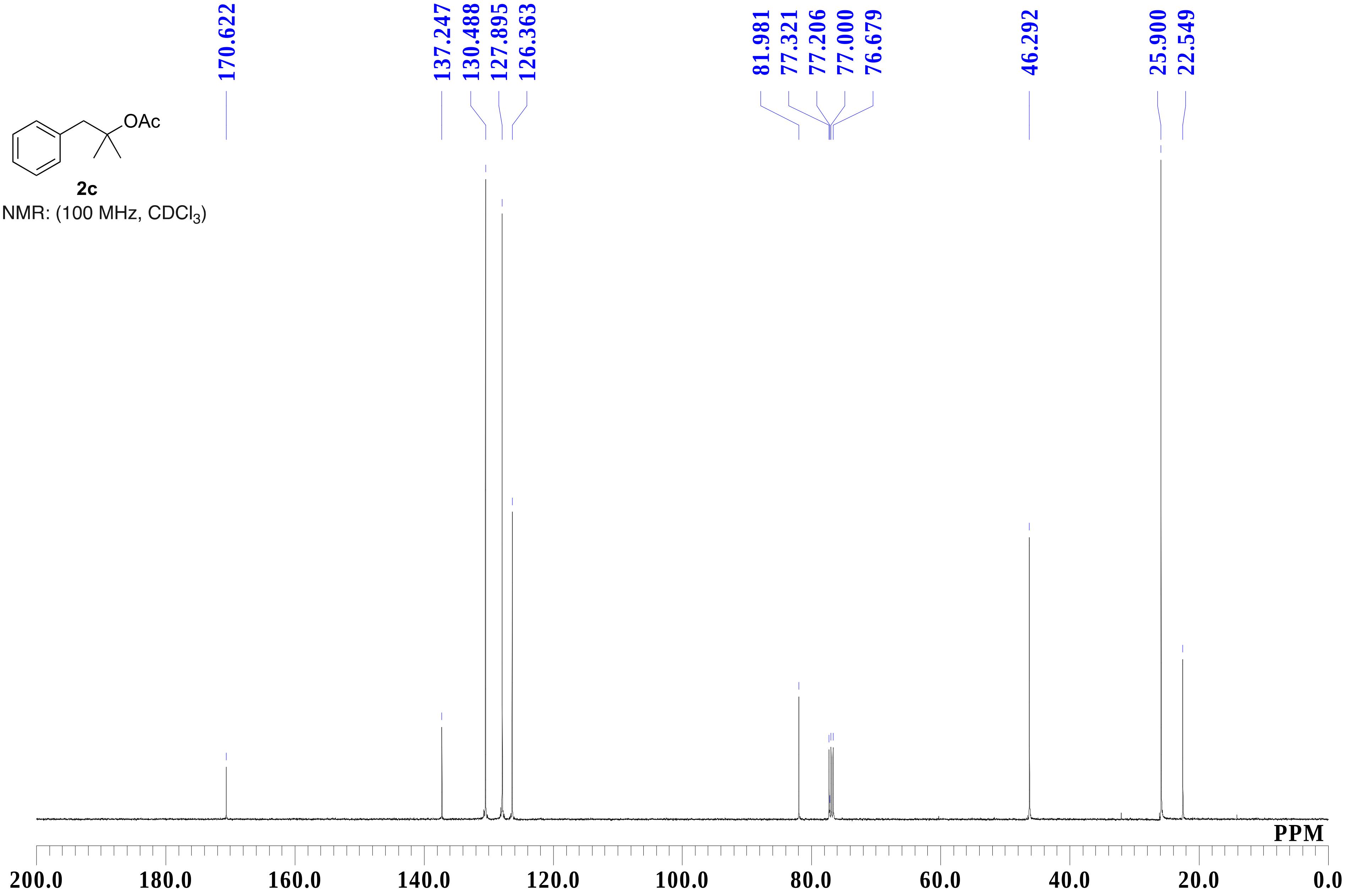

2c

^{13}C NMR: (100 MHz, $CDCl_3$)

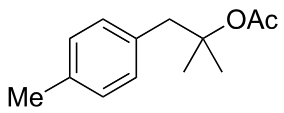

2d

^1H NMR: (400 MHz, $CDCl_3$)

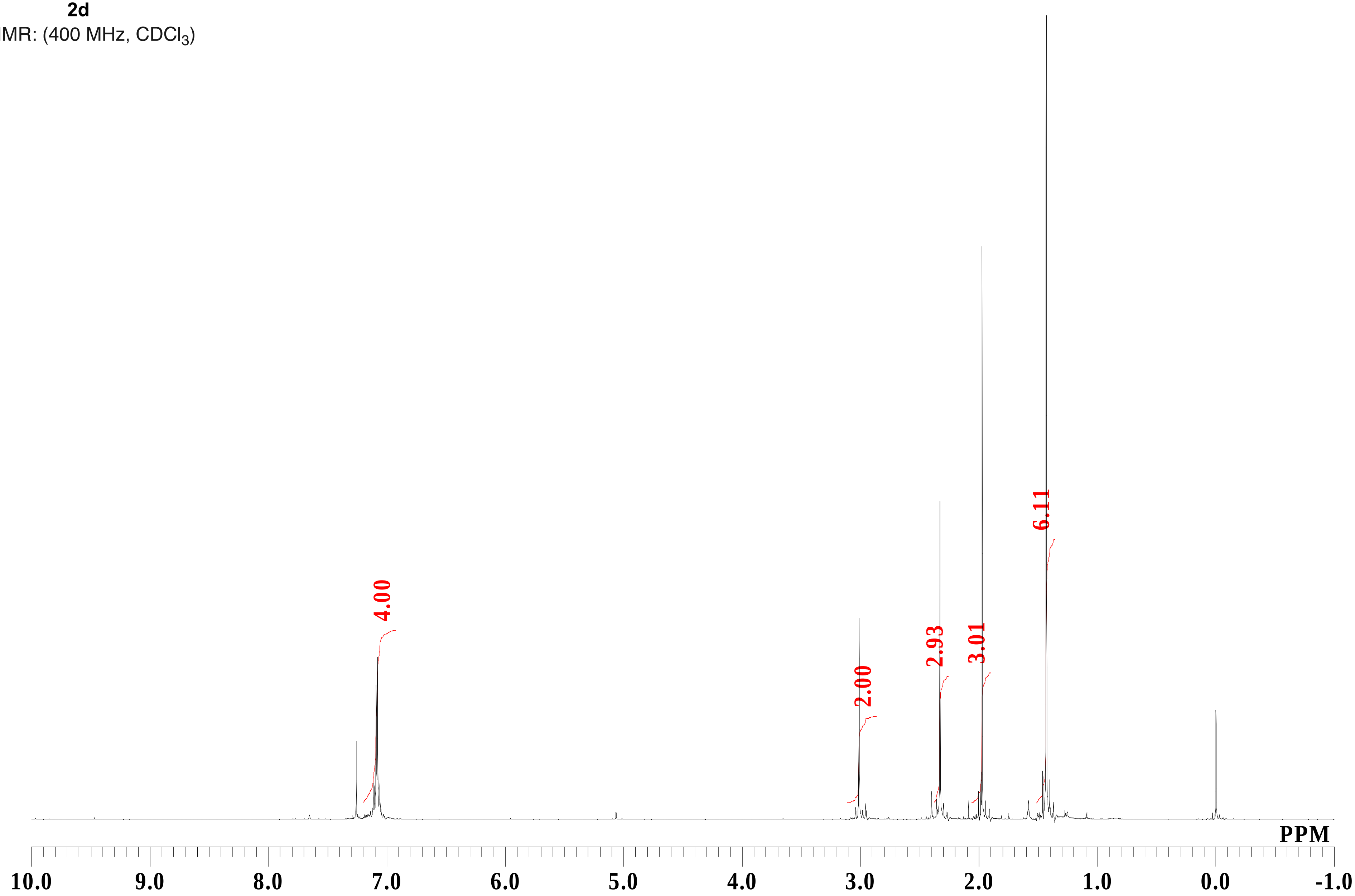

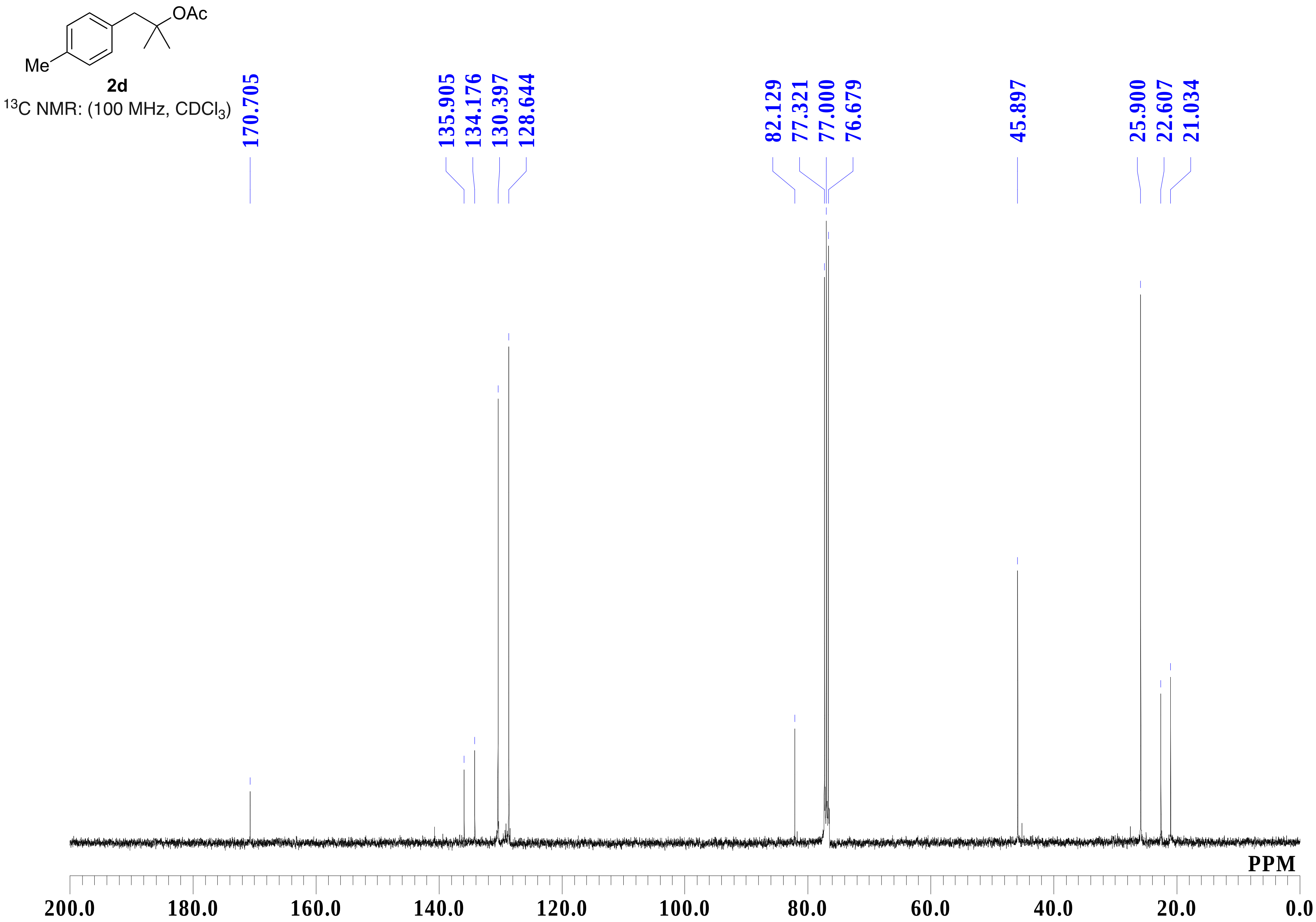
OAc
Me
2d
^{13}C NMR: (100 MHz, CDCl$_3$)
170.705
135.905
134.176
130.397
128.644
82.129
77.321
77.000
76.679
45.897
25.900
22.607
21.034
PPM
200.0
180.0
160.0
140.0
120.0
100.0
80.0
60.0
40.0
20.0
0.0

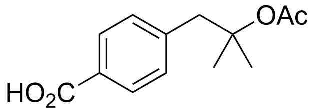

2e

^1H NMR: (400 MHz, $CDCl_3$)

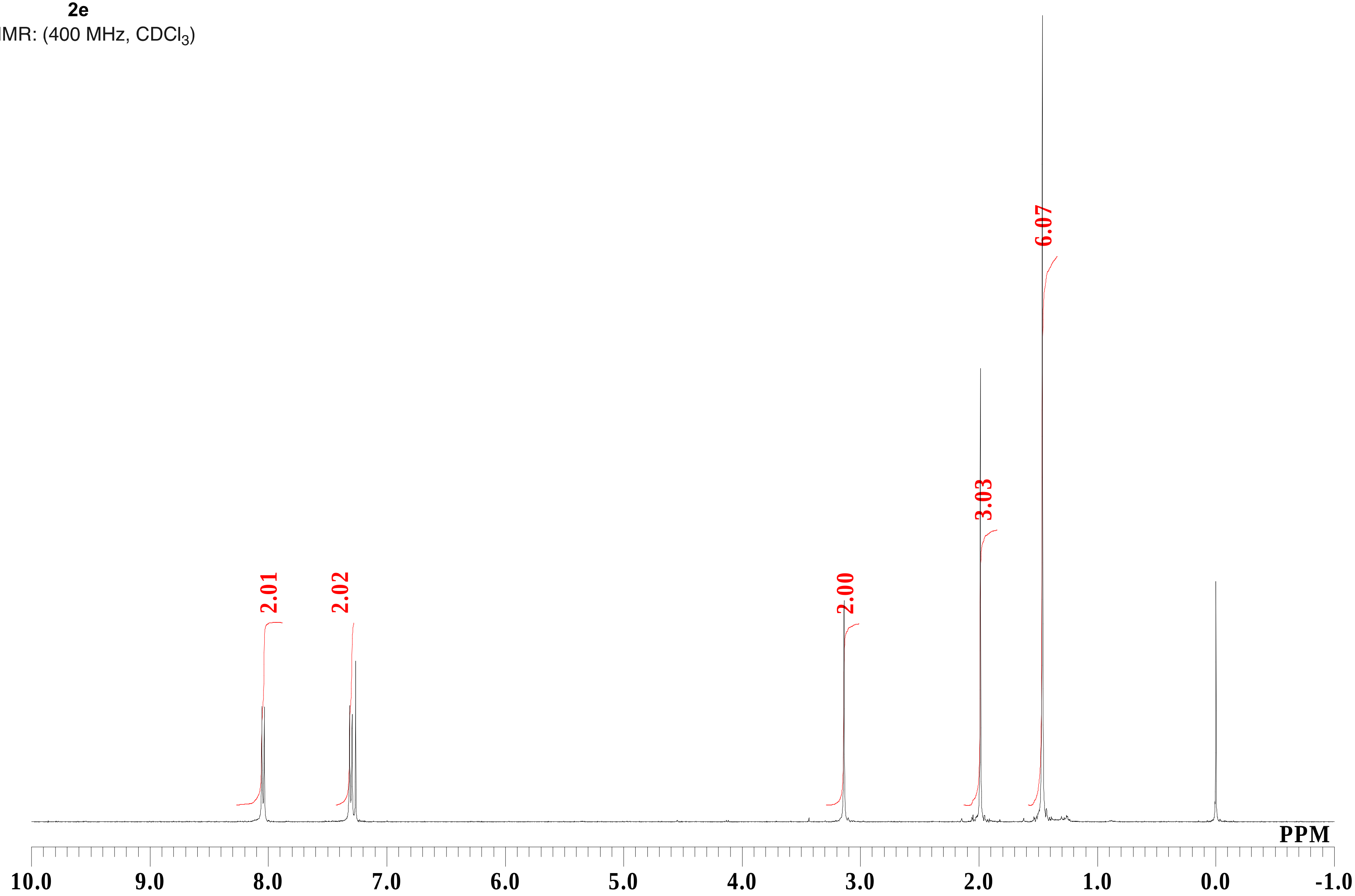

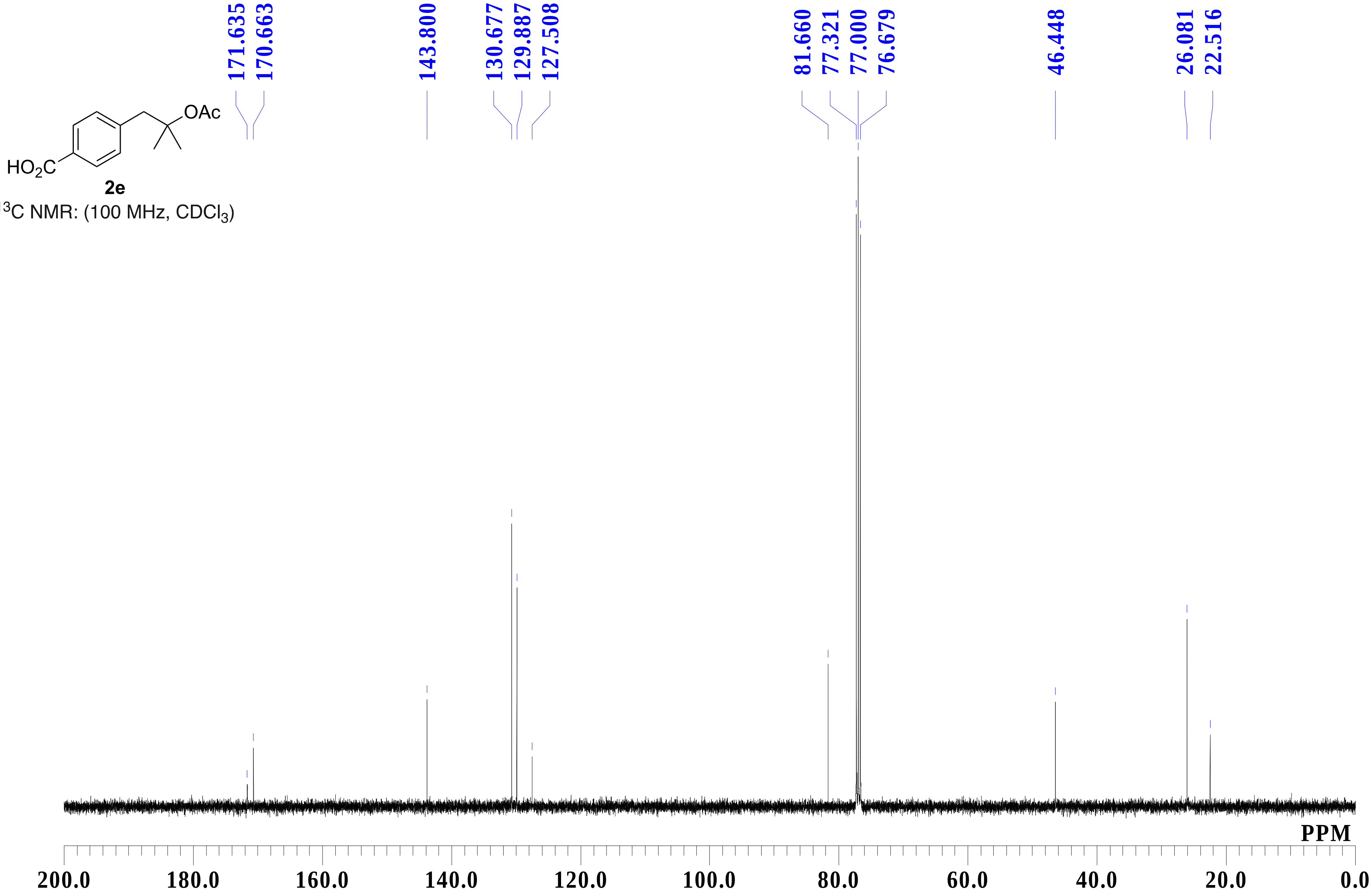

2e

^{13}C NMR: (100 MHz, $CDCl_3$)

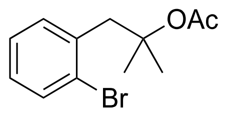

2f

^{1}H NMR: (400 MHz, $CDCl_3$)

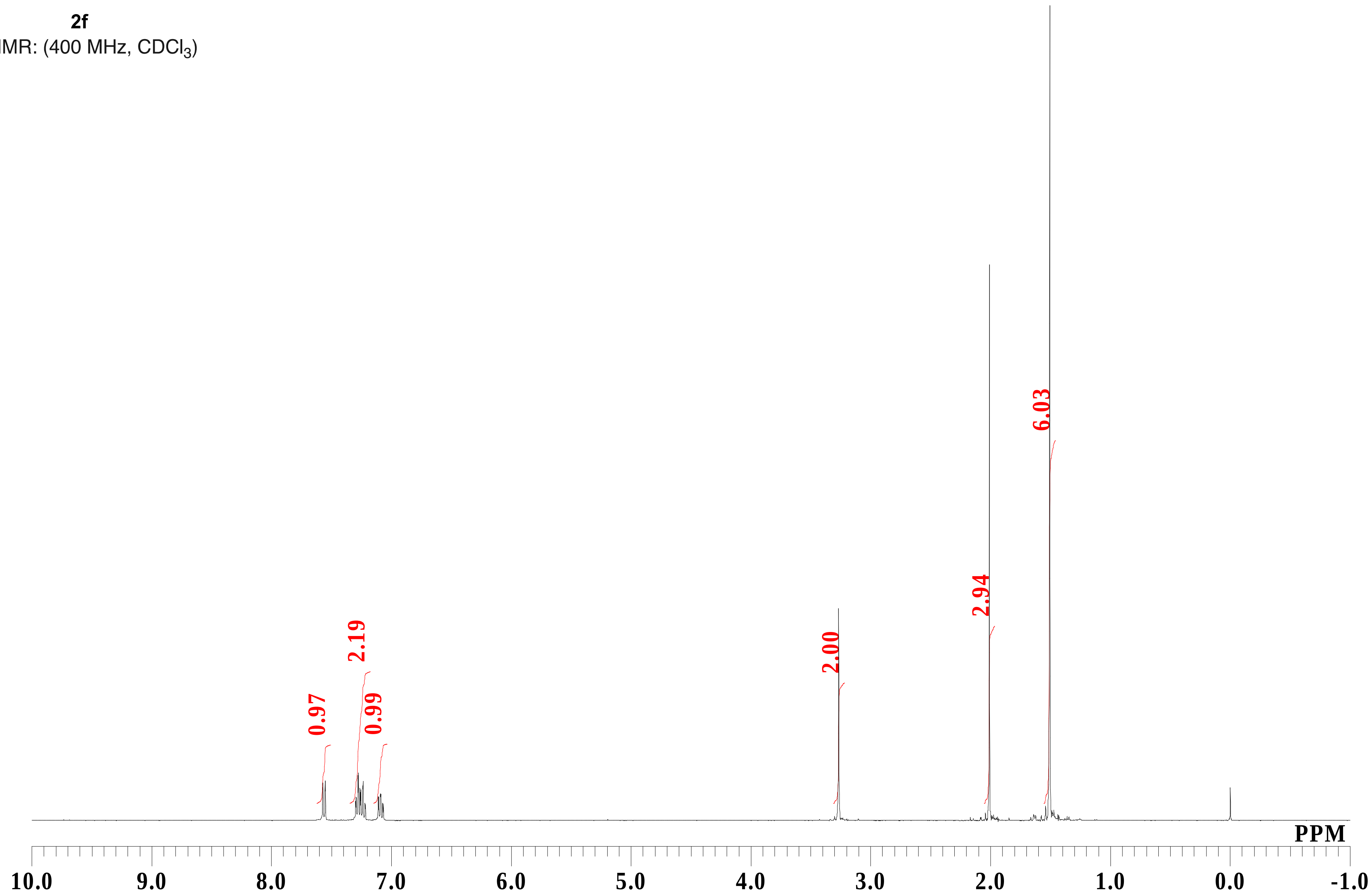

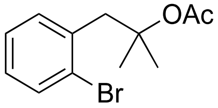

2f

^{13}C NMR: (100 MHz, $CDCl_3$)

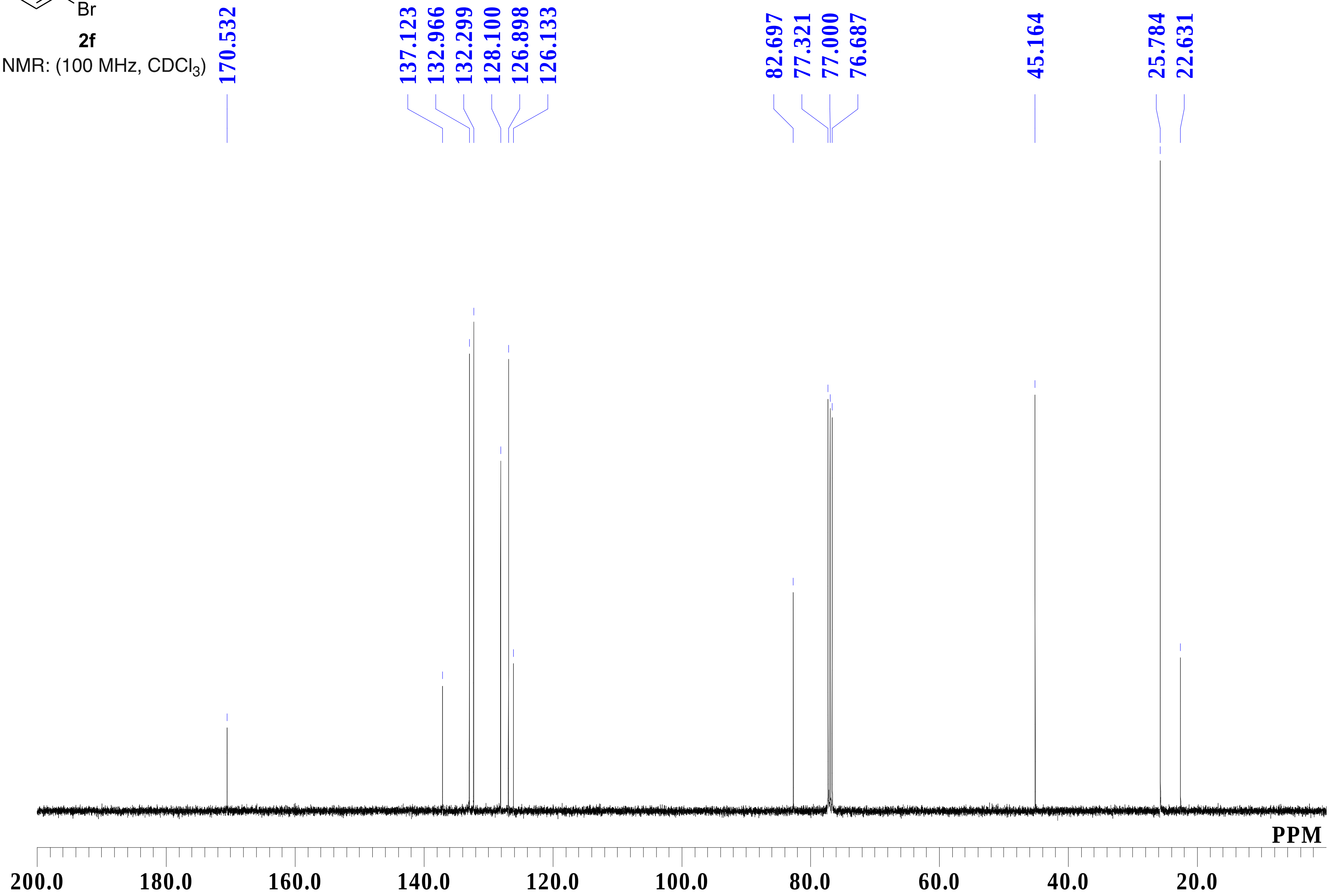

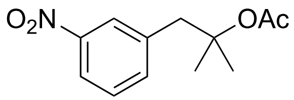

2g

^{1}H NMR: (400 MHz, $CDCl_3$)

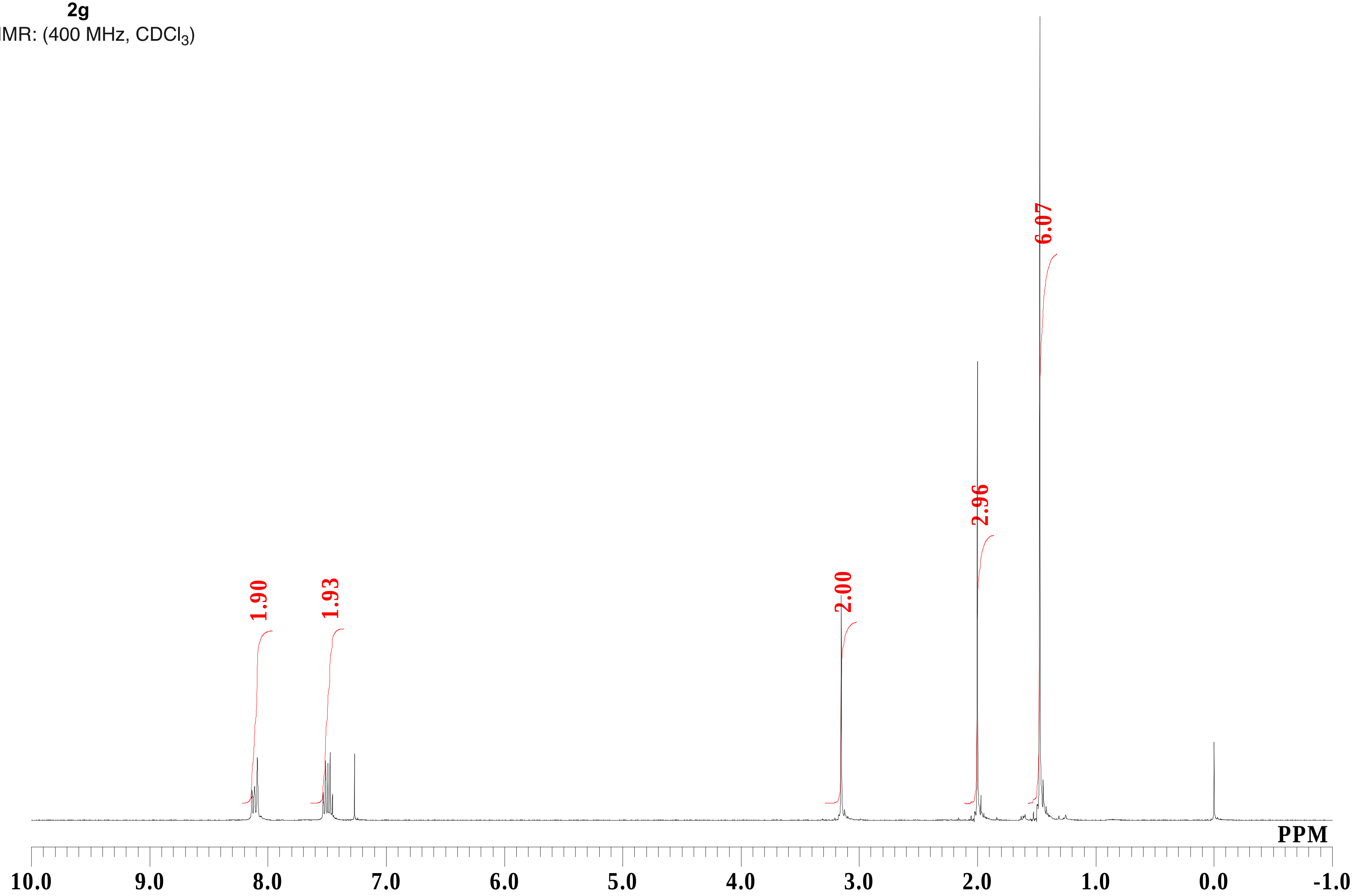

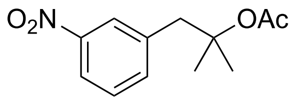

2g

^{13}C NMR: (100 MHz, $CDCl_3$)

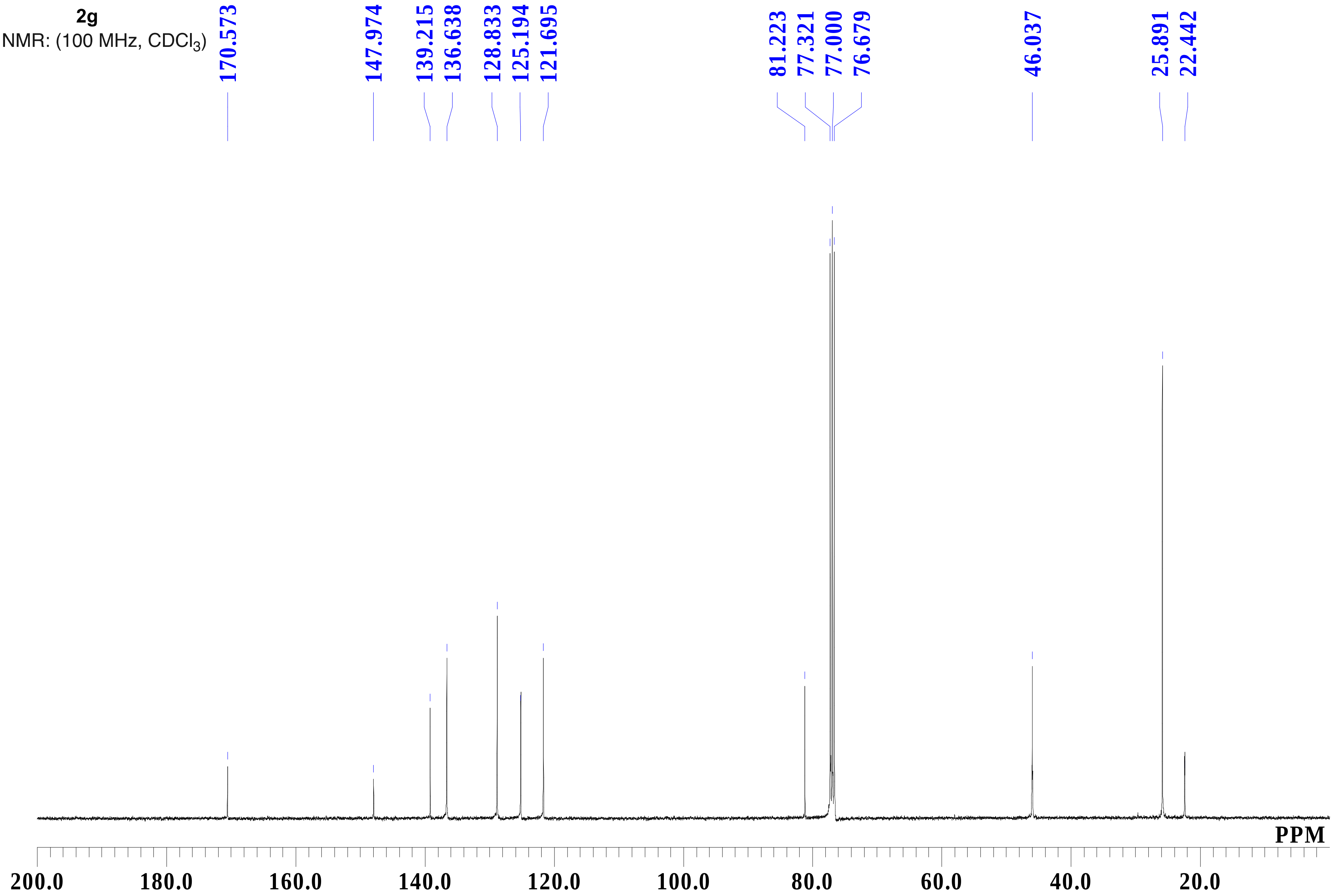

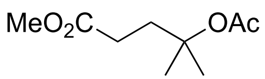

2h

^{1}H NMR: (400 MHz, $CDCl_3$)

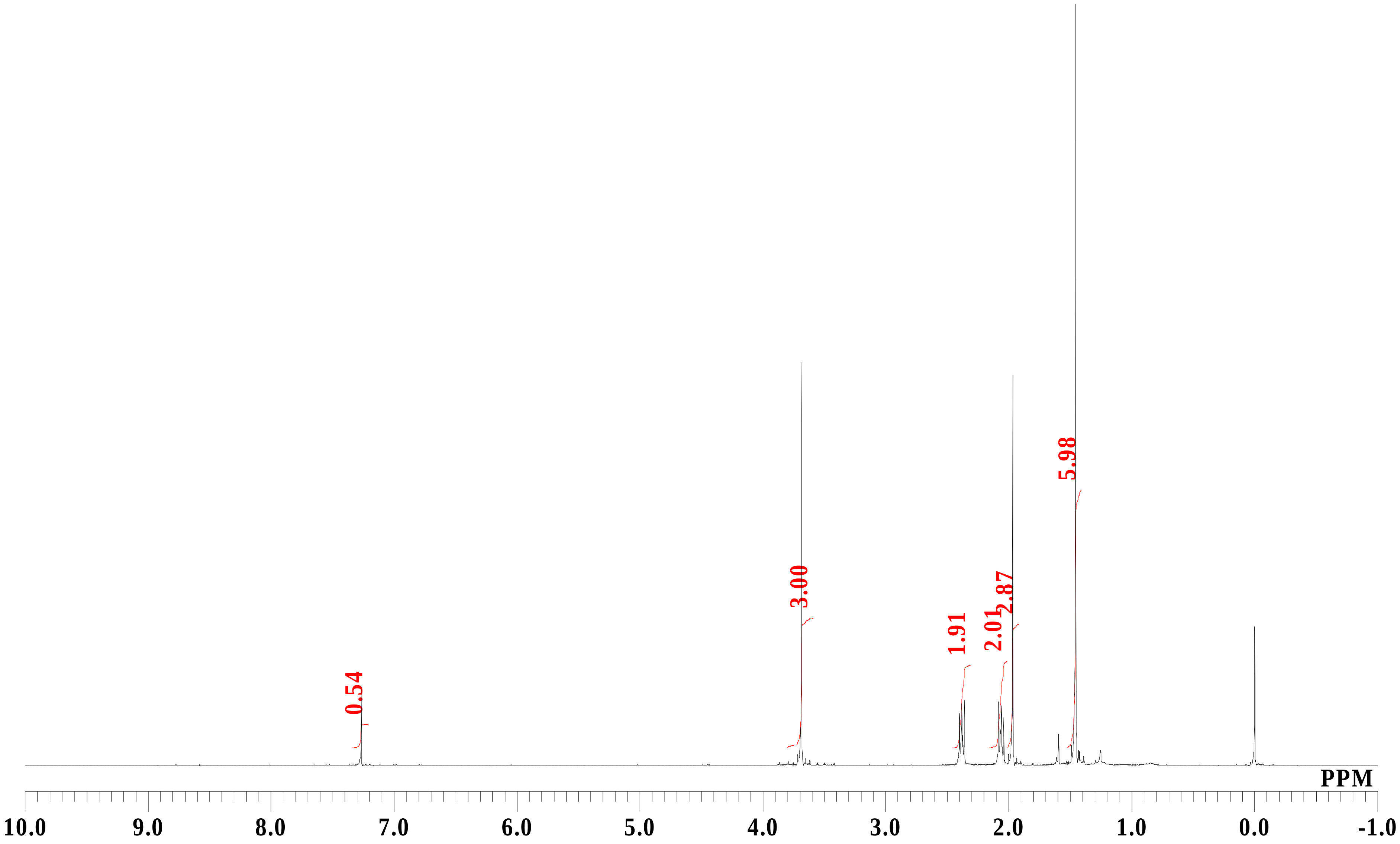

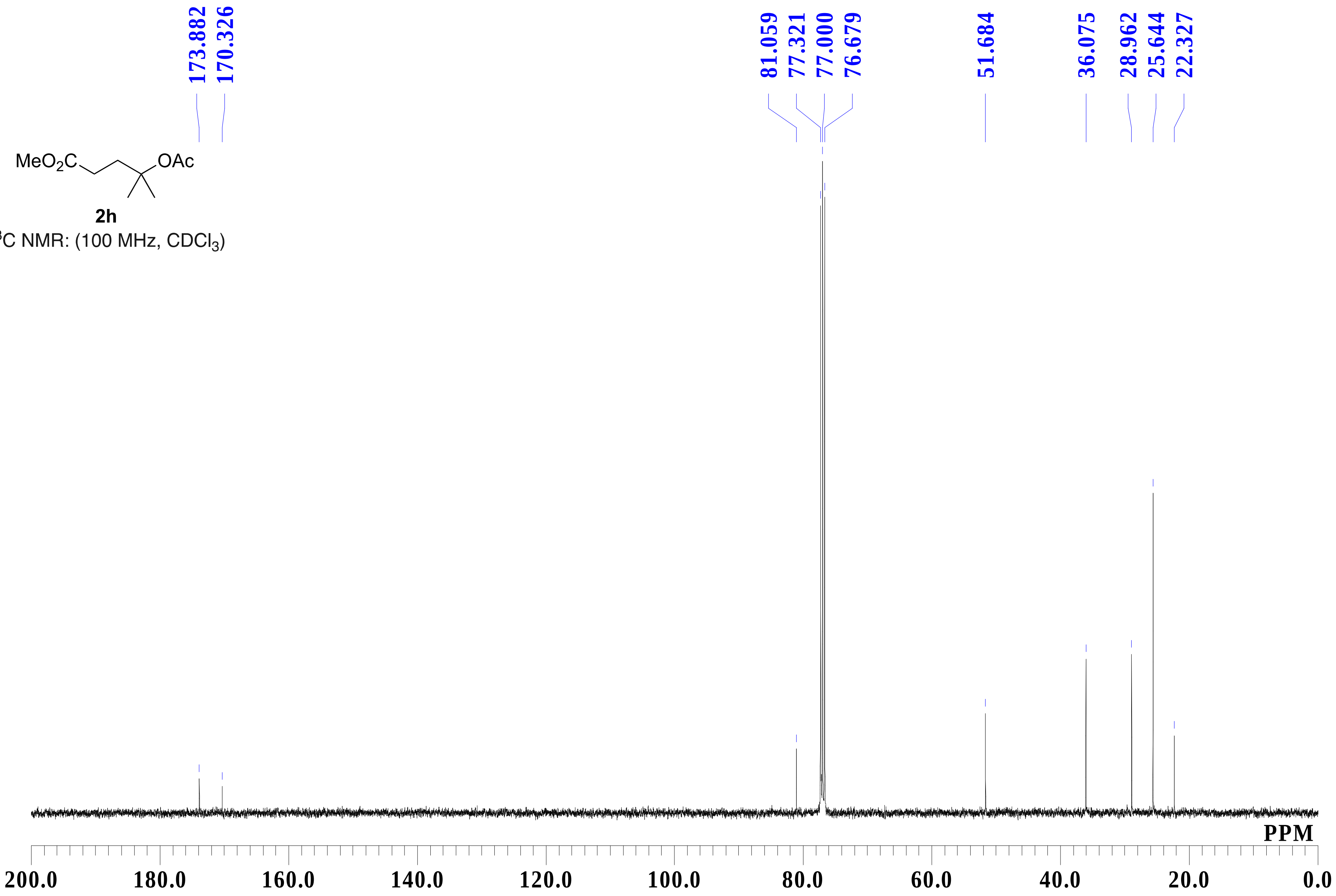

2h

^{13}C NMR: (100 MHz, $CDCl_3$)

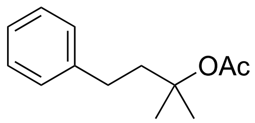

2i

^{1}H NMR: (400 MHz, $CDCl_3$)

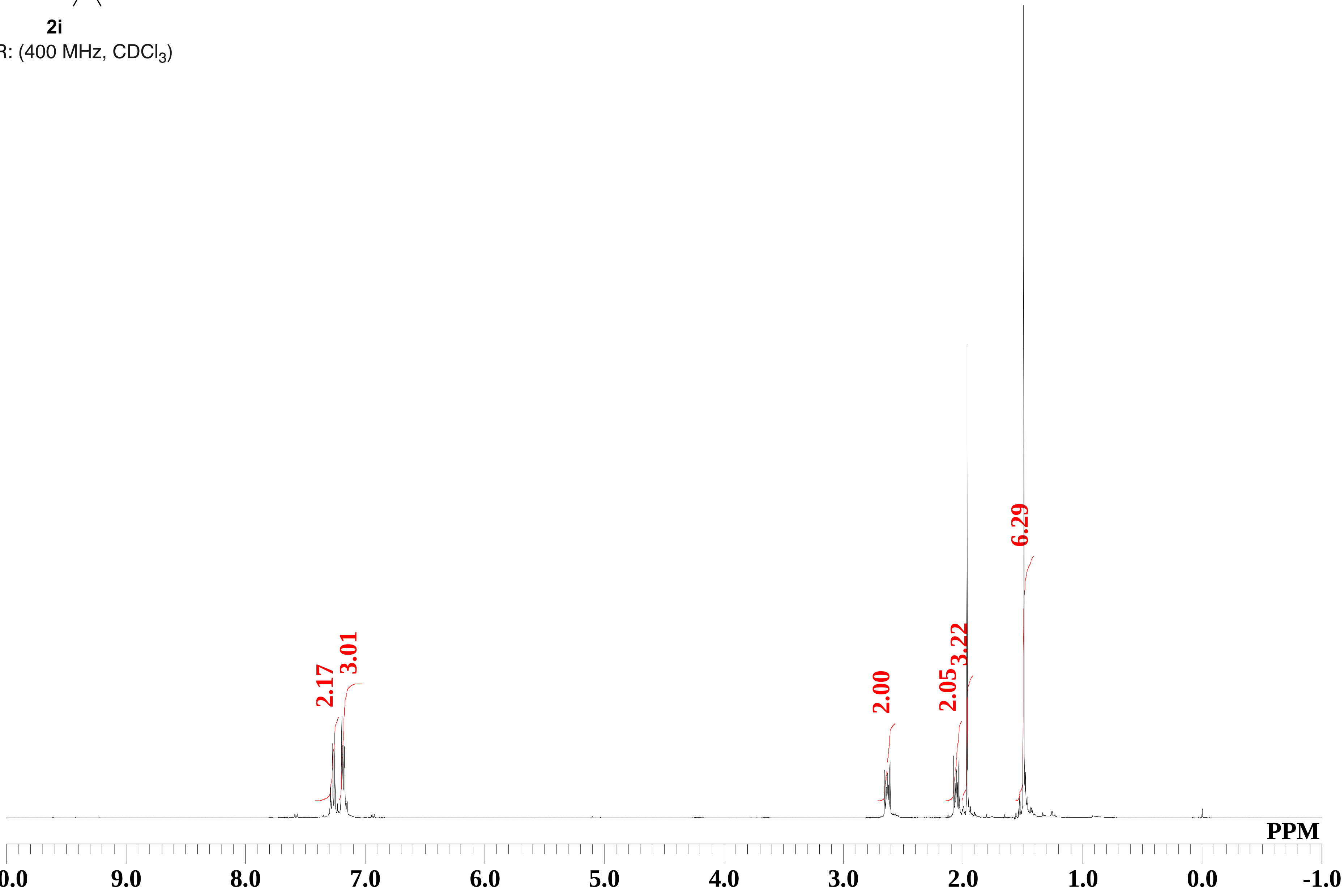

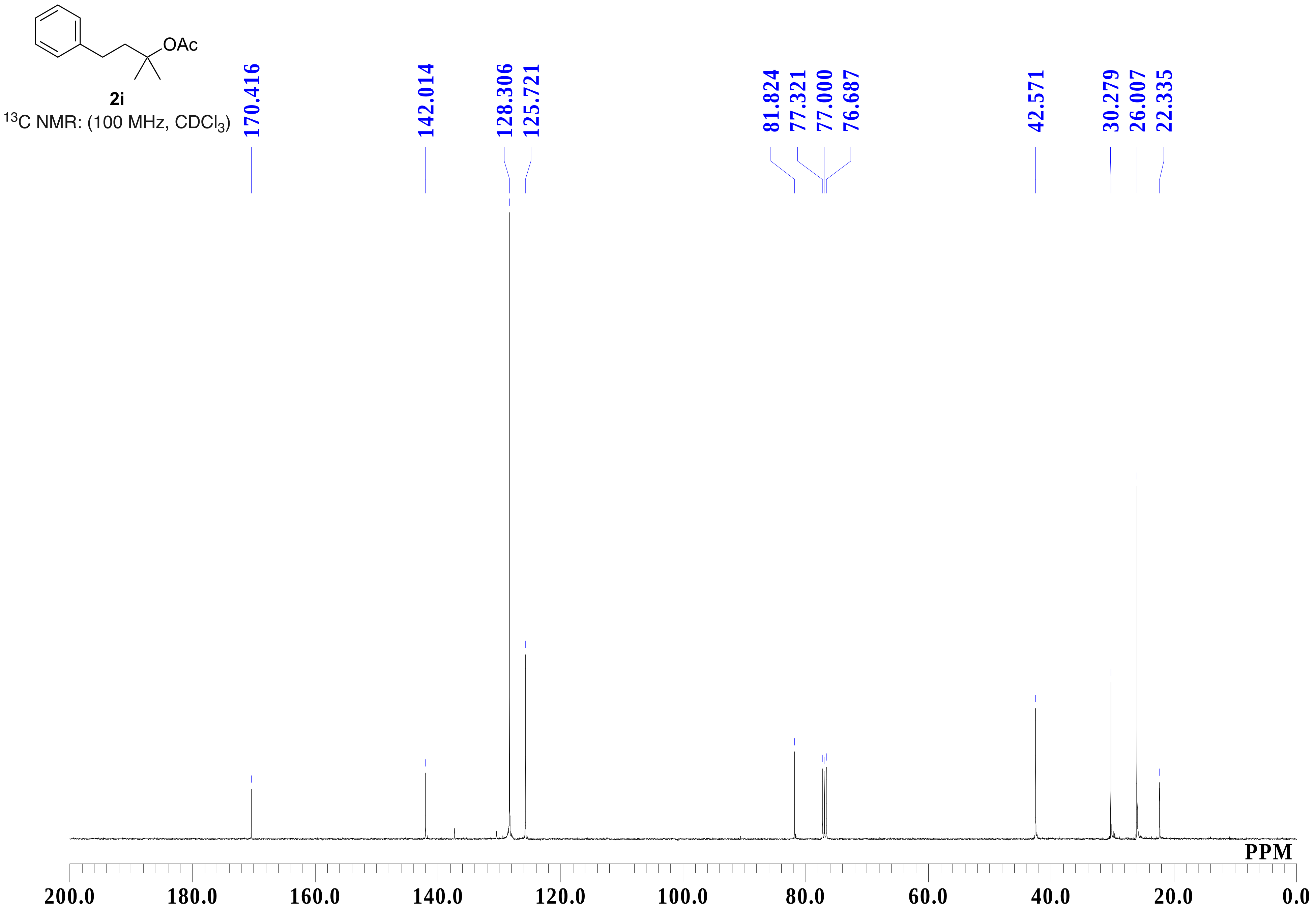
OAc
2i
13C NMR: (100 MHz, CDCl3)
170.416
142.014
128.306
125.721
81.824
77.321
77.000
76.687
42.571
30.279
26.007
22.335
PPM
200.0
180.0
160.0
140.0
120.0
100.0
80.0
60.0
40.0
20.0
0.0

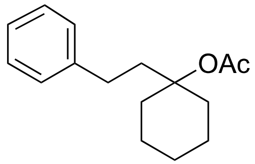

2j

^{1}H NMR: (400 MHz, $CDCl_3$)

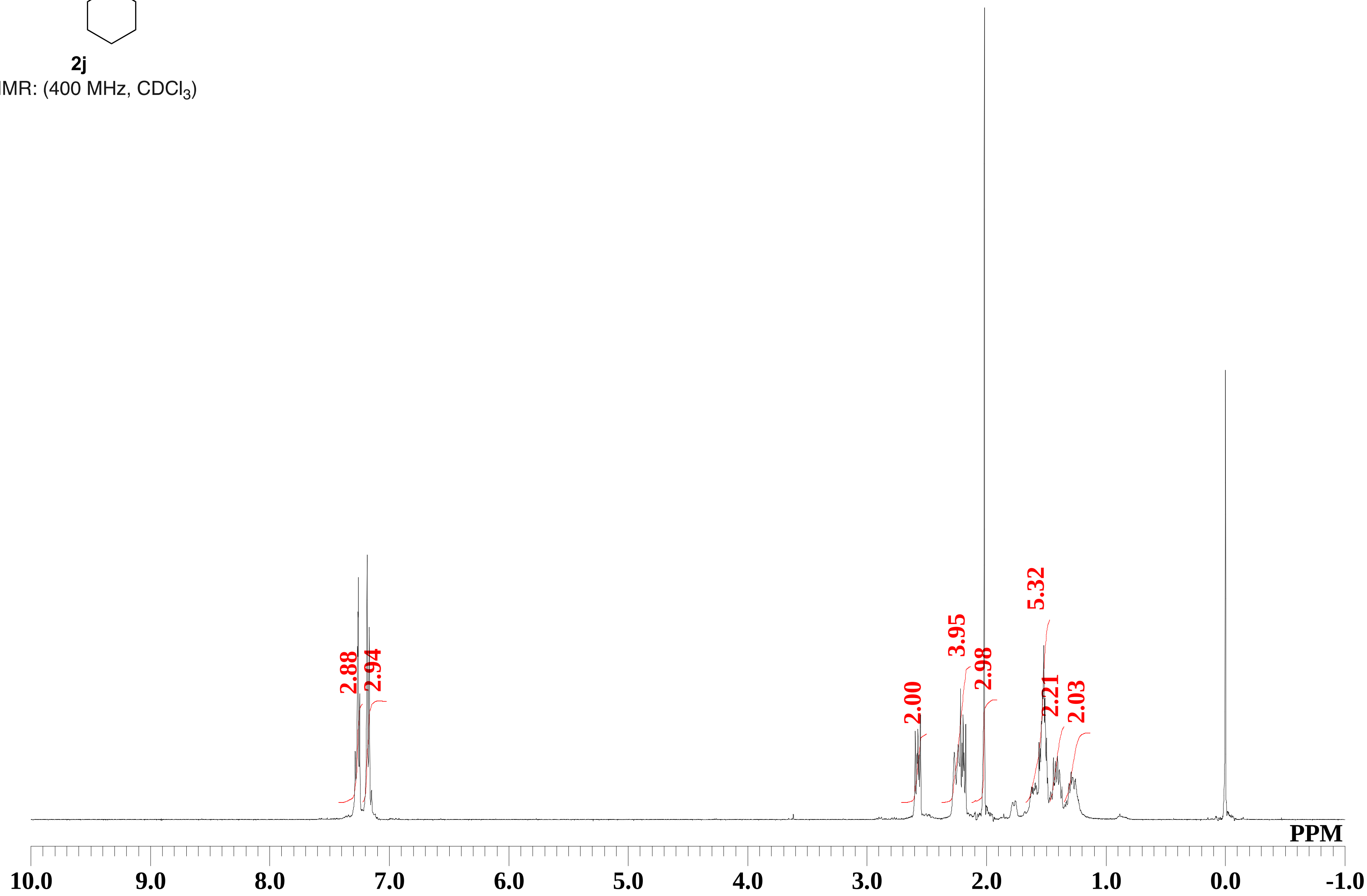

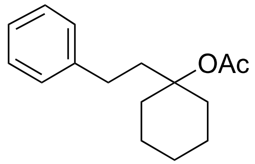

2j

^{13}C NMR: (100 MHz, $CDCl_3$)

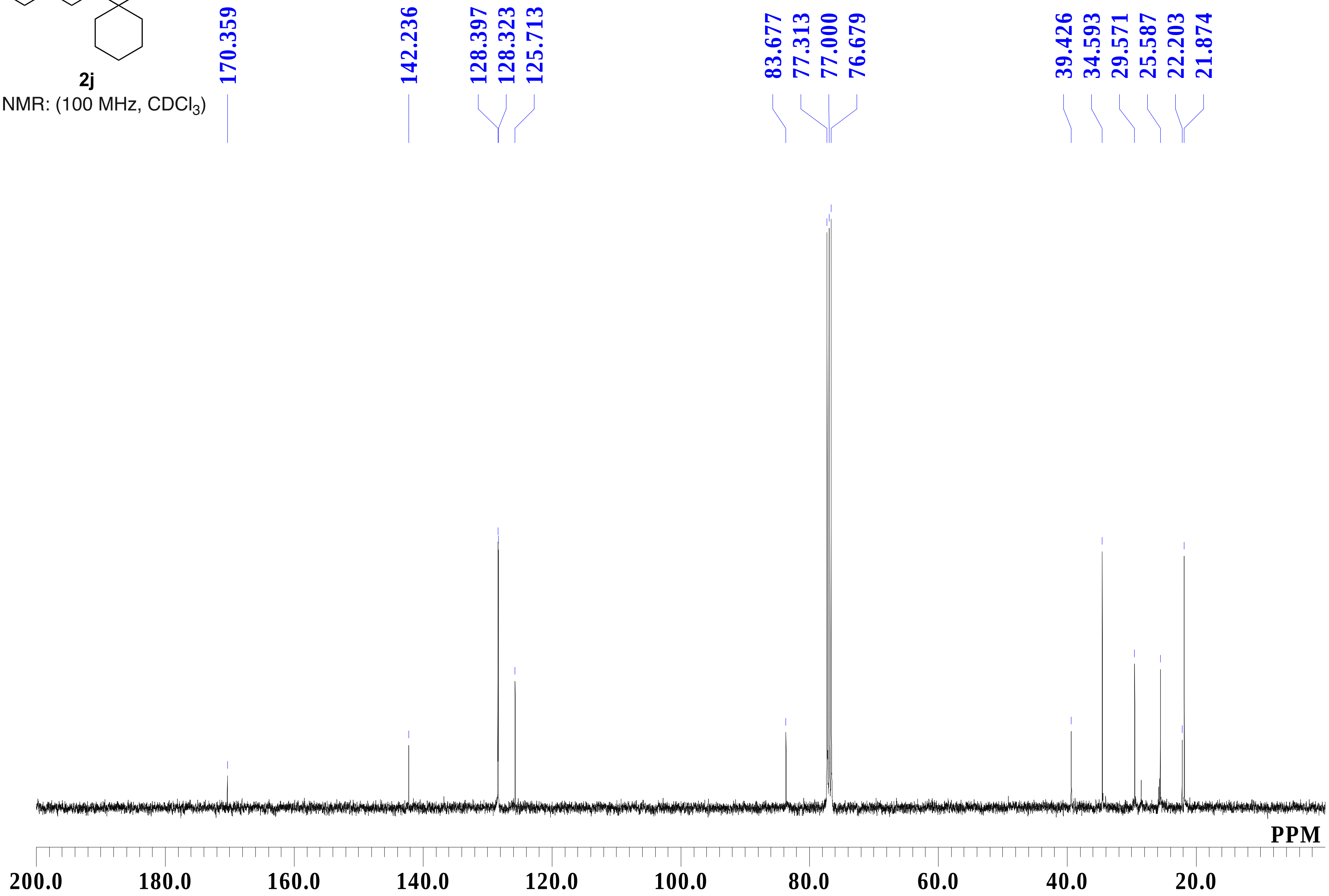

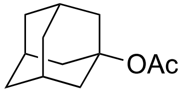

2k

^{1}H NMR: (400 MHz, $CDCl_3$)

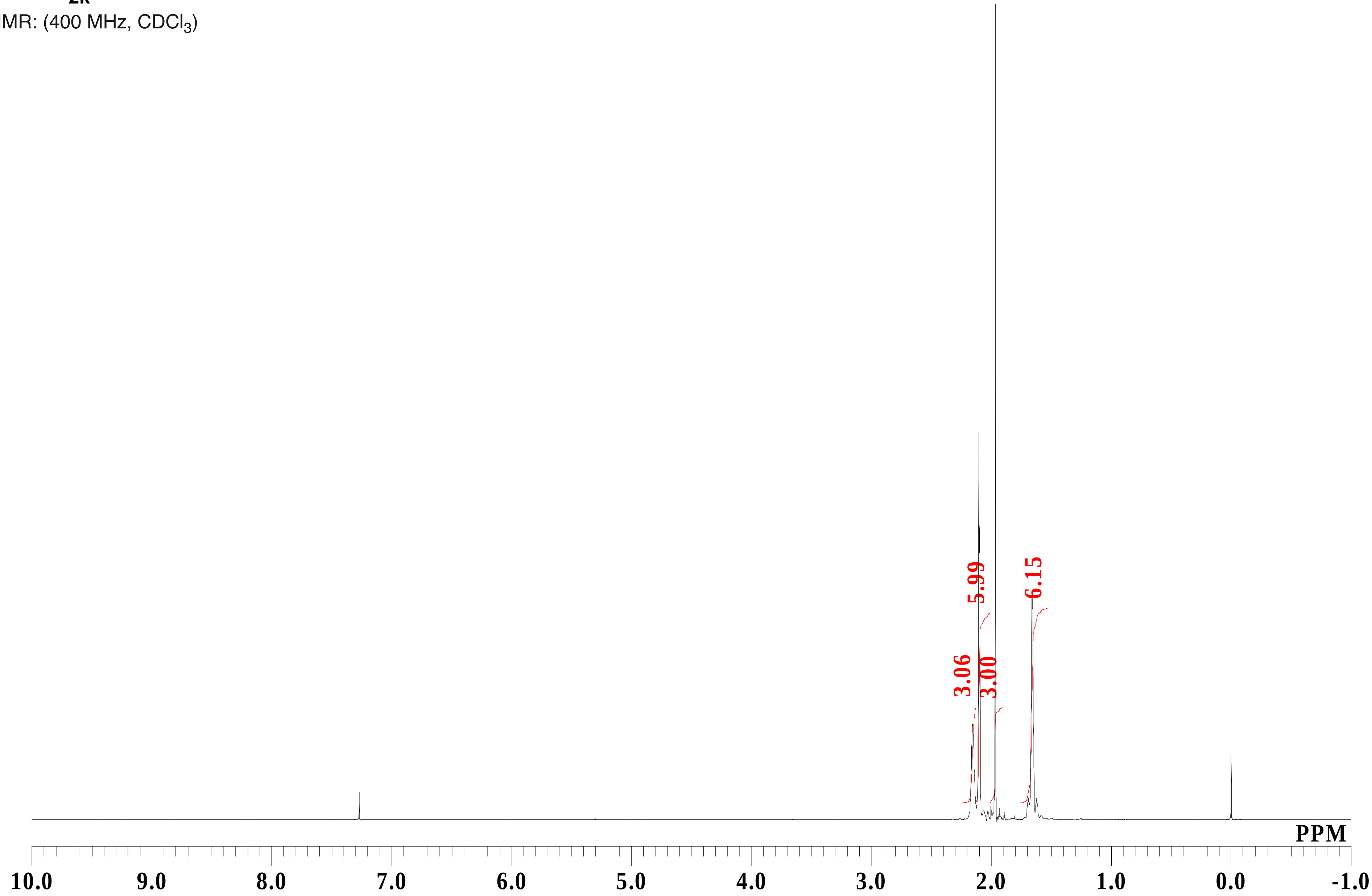

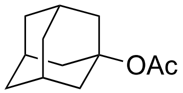

2k

^{13}C NMR: (100 MHz, $CDCl_3$)

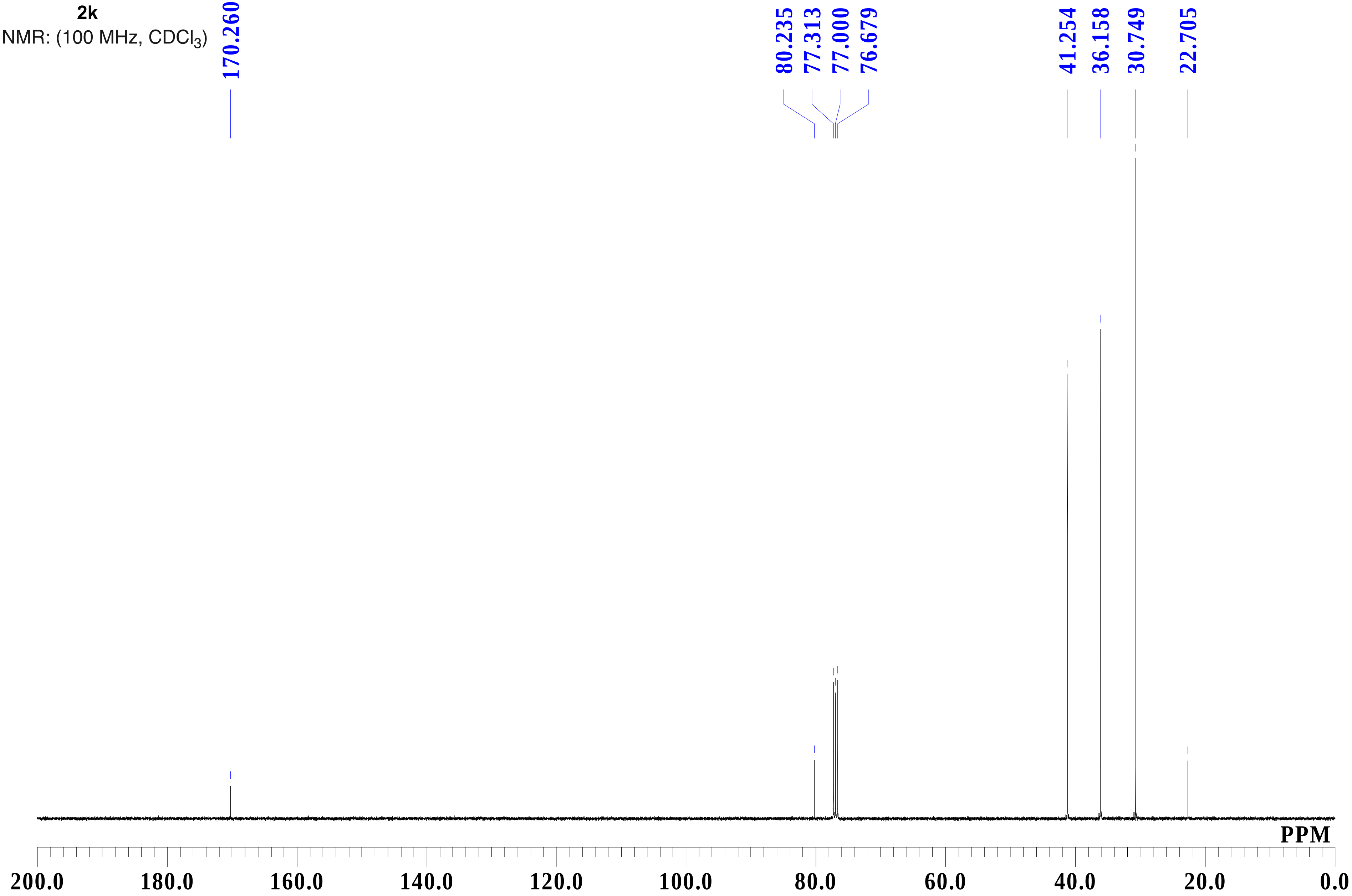

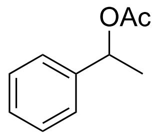

2l

^{1}H NMR: (400 MHz, $CDCl_3$)

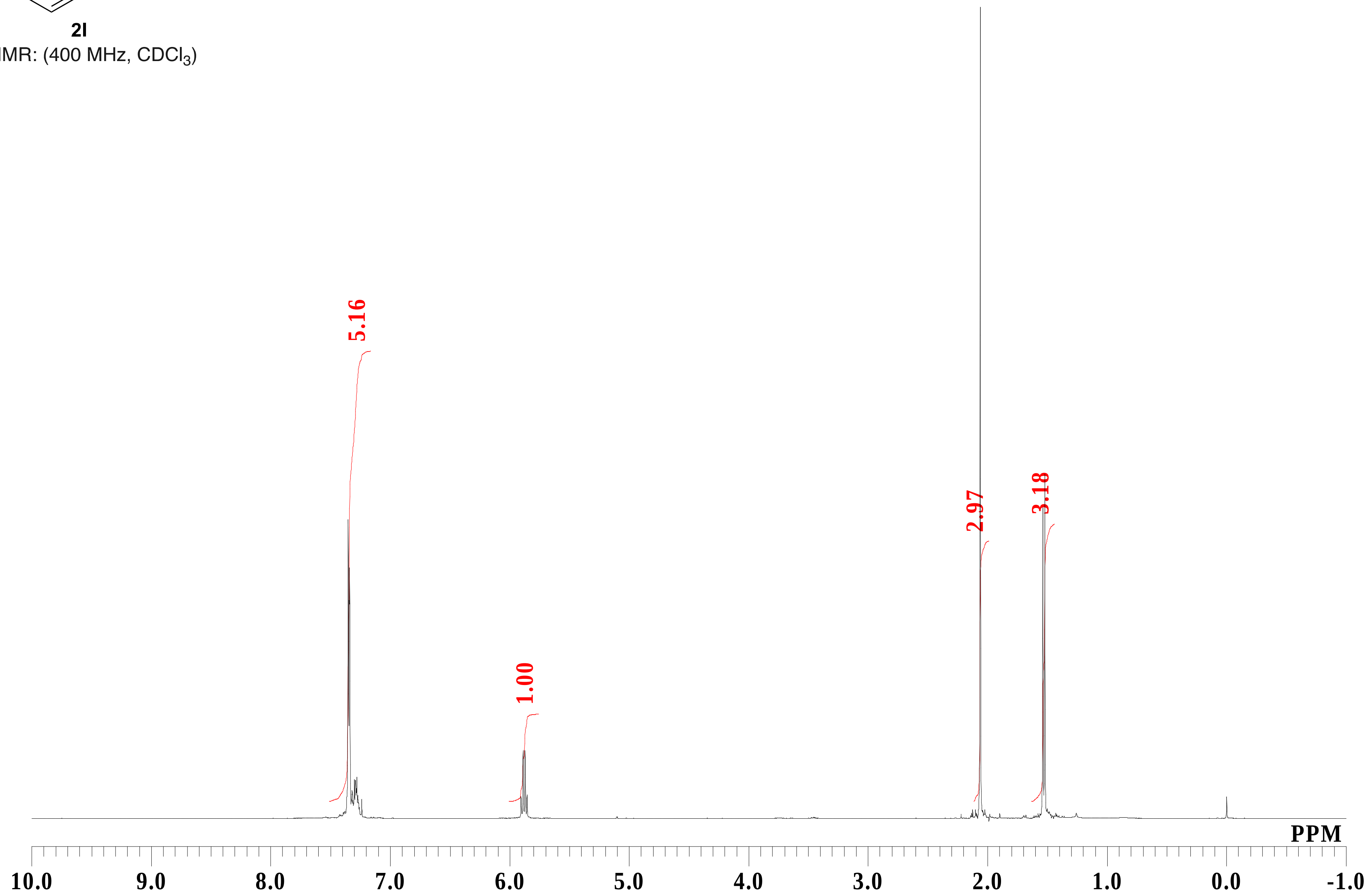

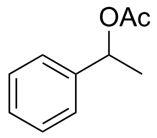

2l

^{13}C NMR: (100 MHz, $CDCl_3$)

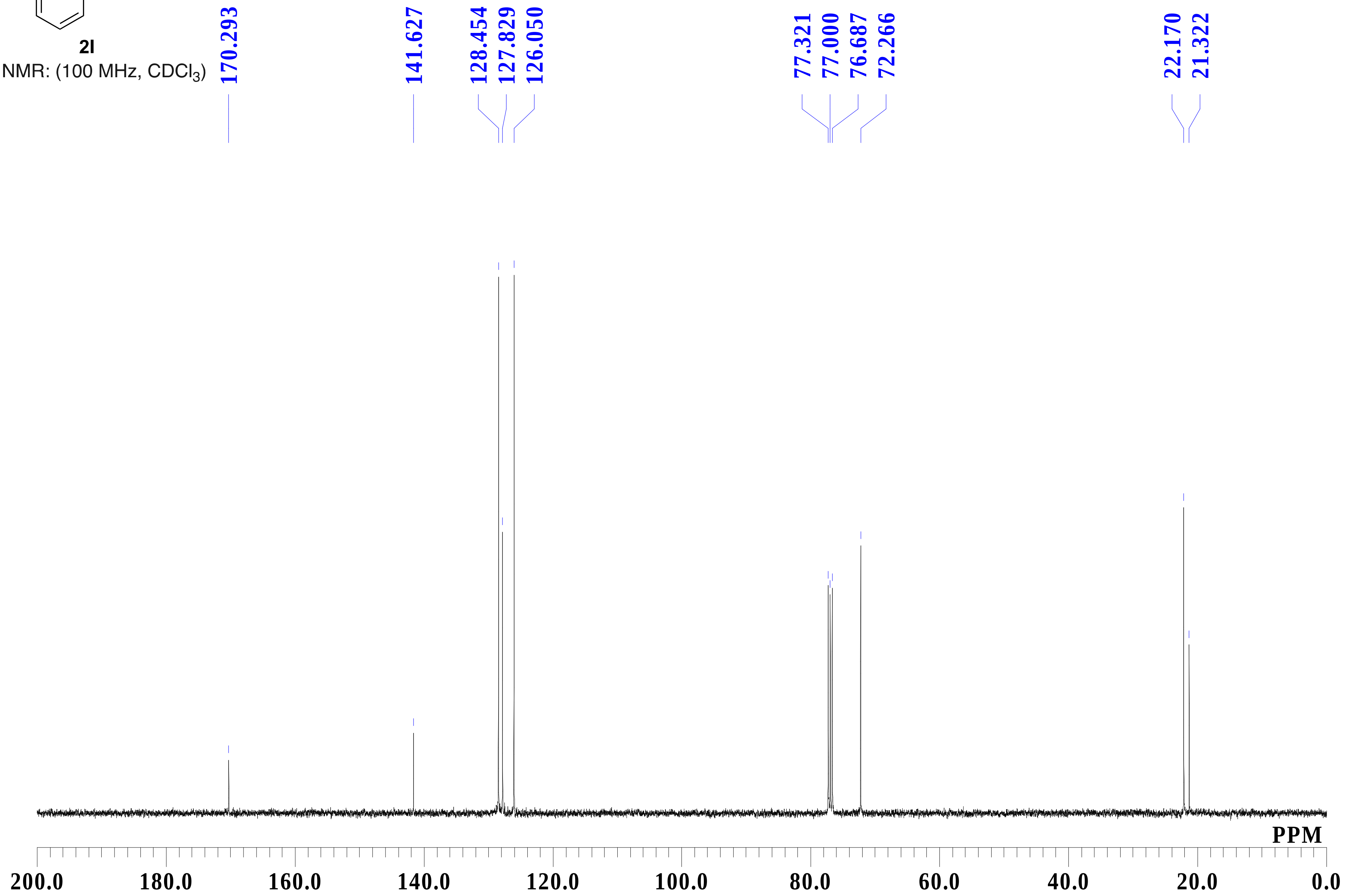

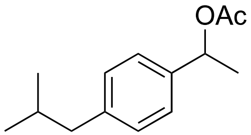

2m

^{1}H NMR: (400 MHz, $CDCl_3$)

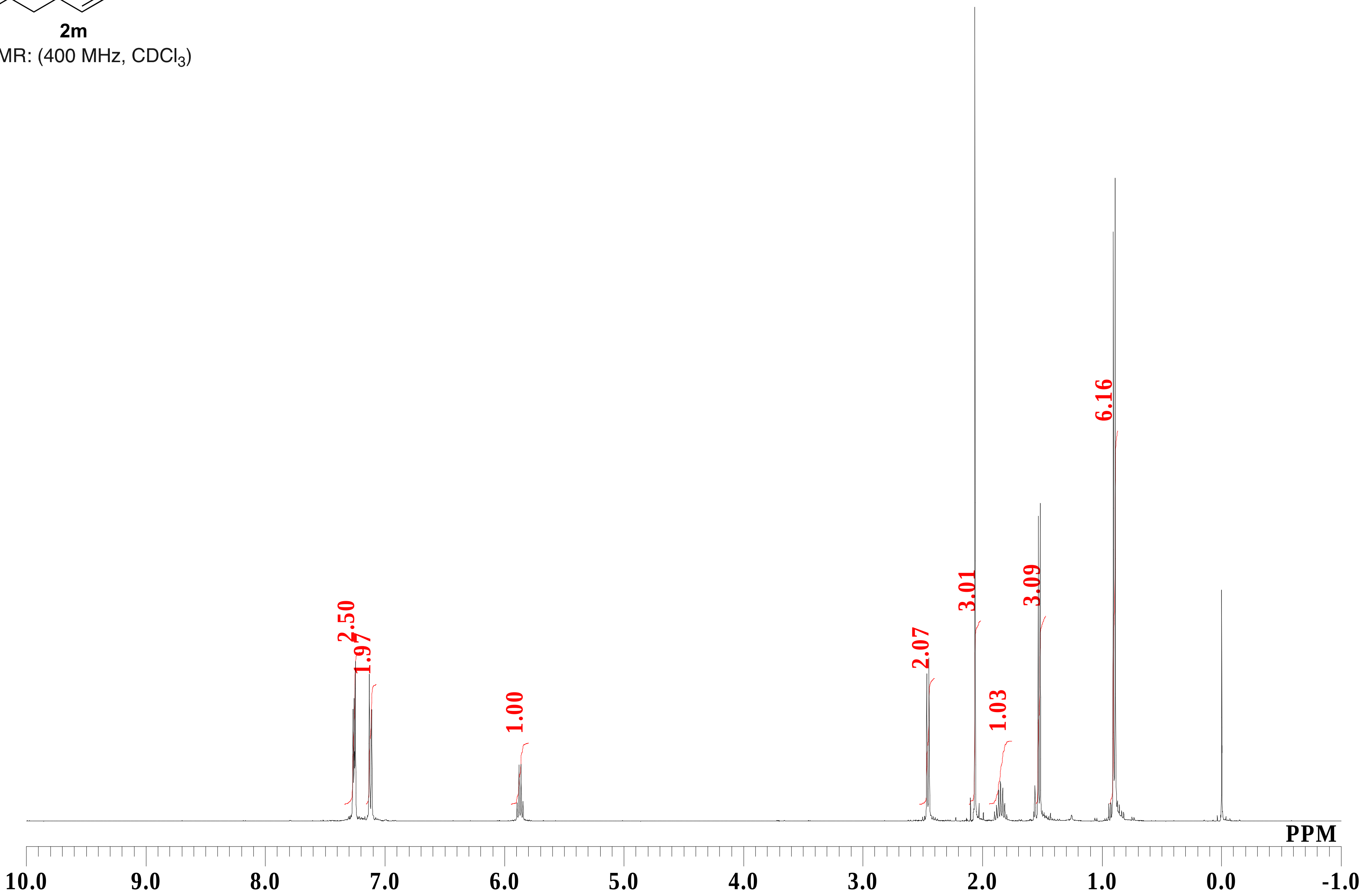

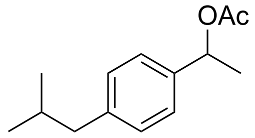

2m

^{13}C NMR: (100 MHz, $CDCl_3$)

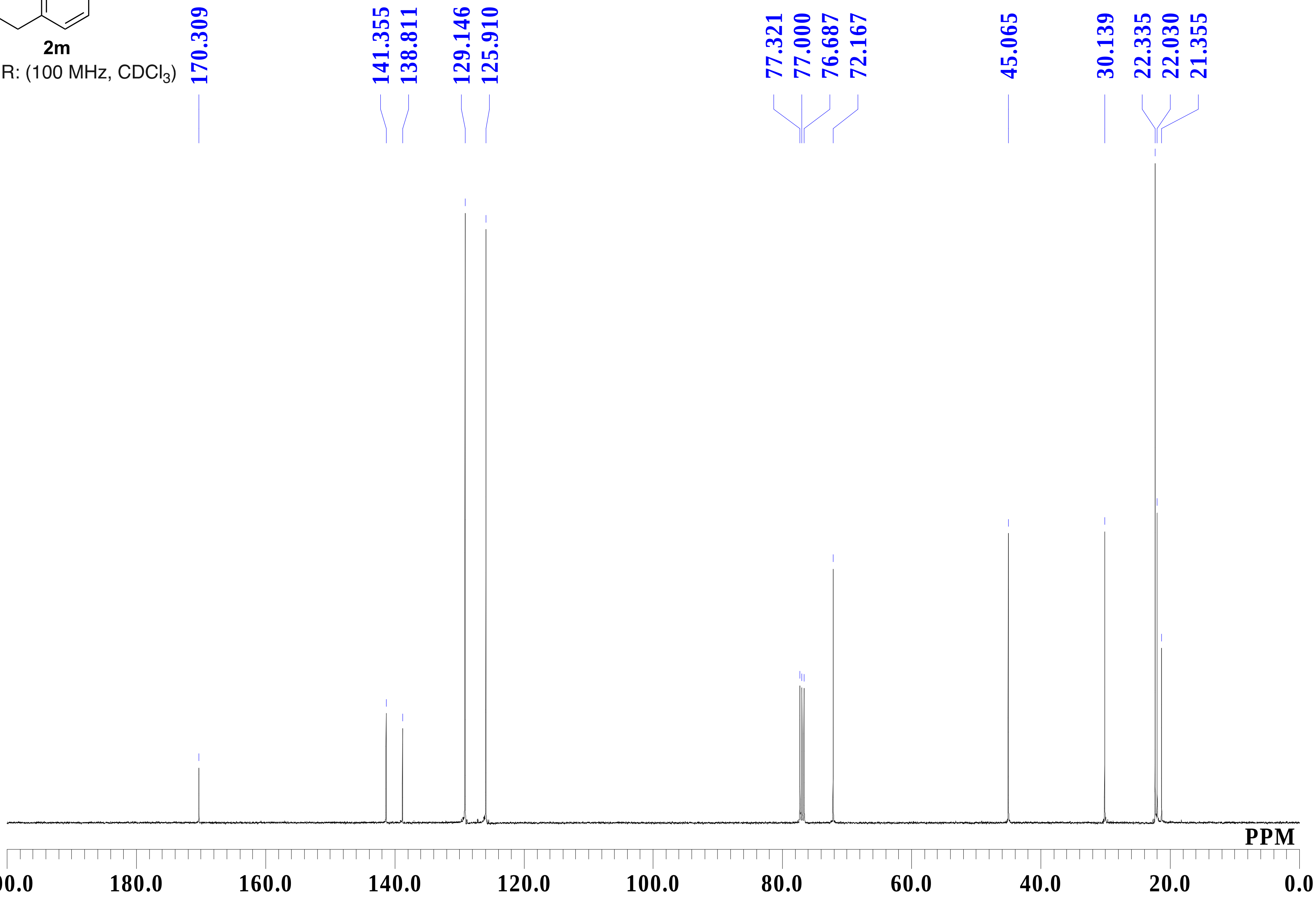

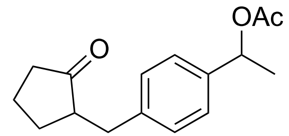

2n

^{1}H NMR: (400 MHz, $CDCl_3$)

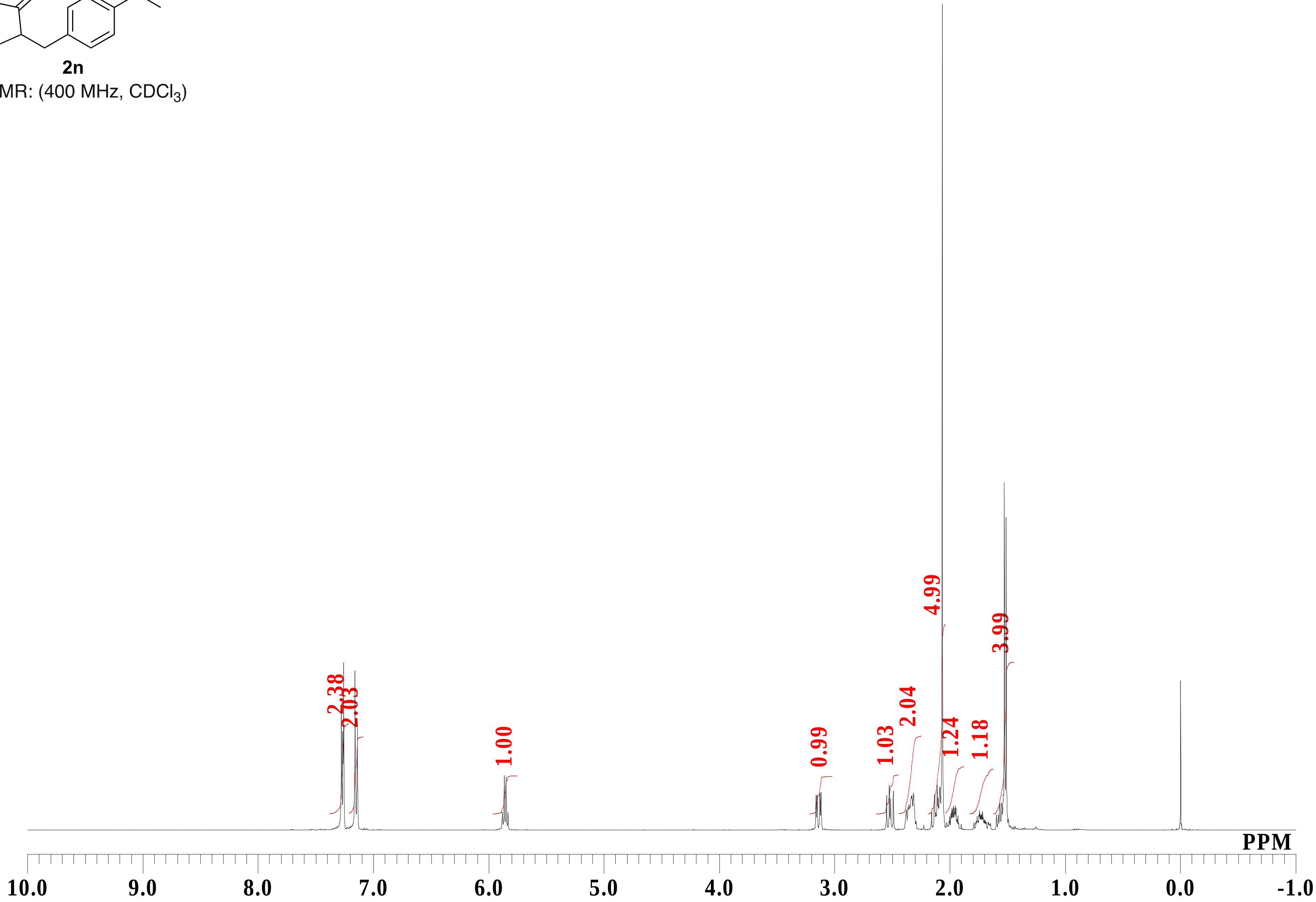

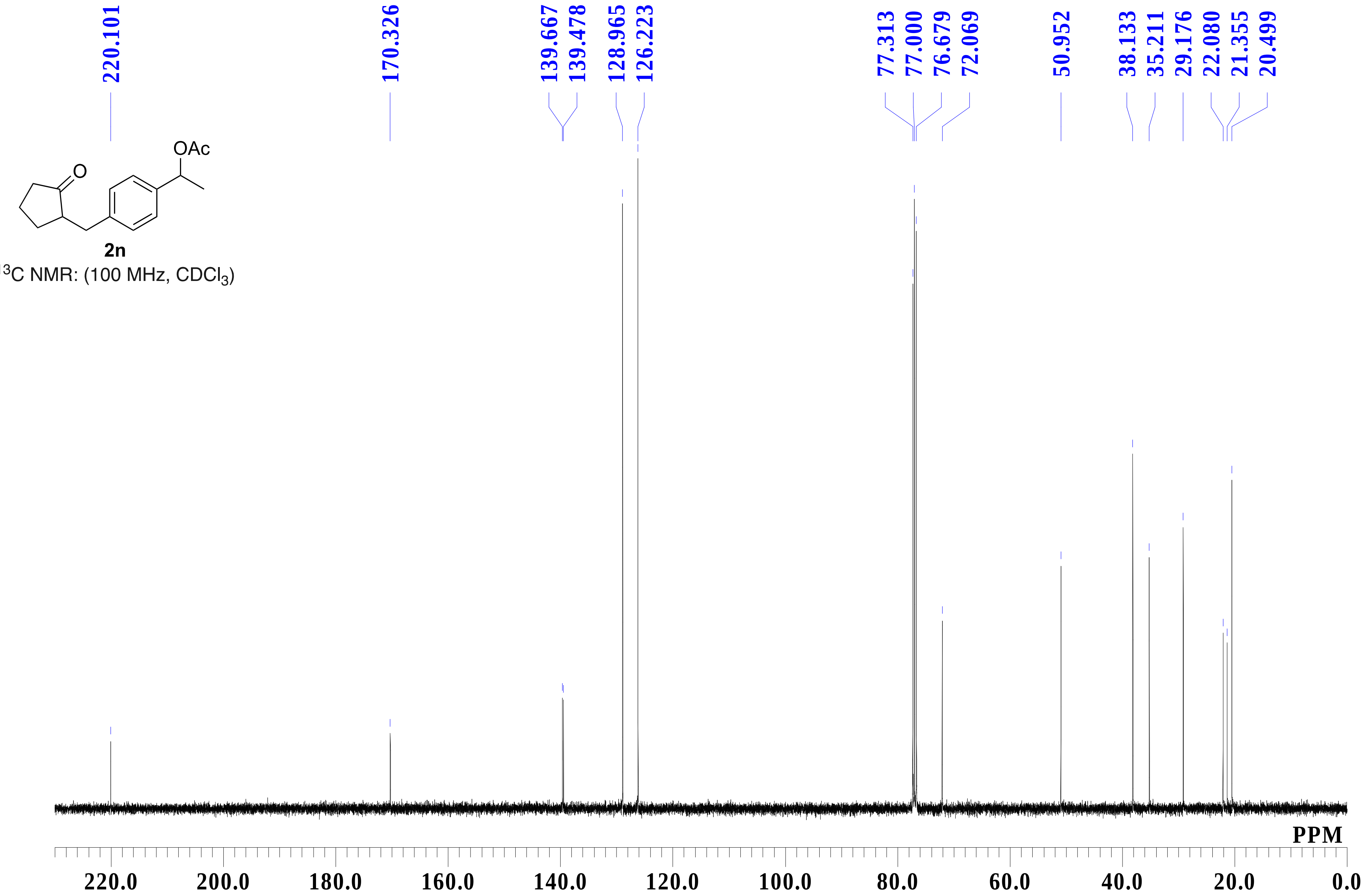

2n

^{13}C NMR: (100 MHz, $CDCl_3$)

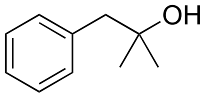

3c

^{1}H NMR: (400 MHz, $CDCl_3$)

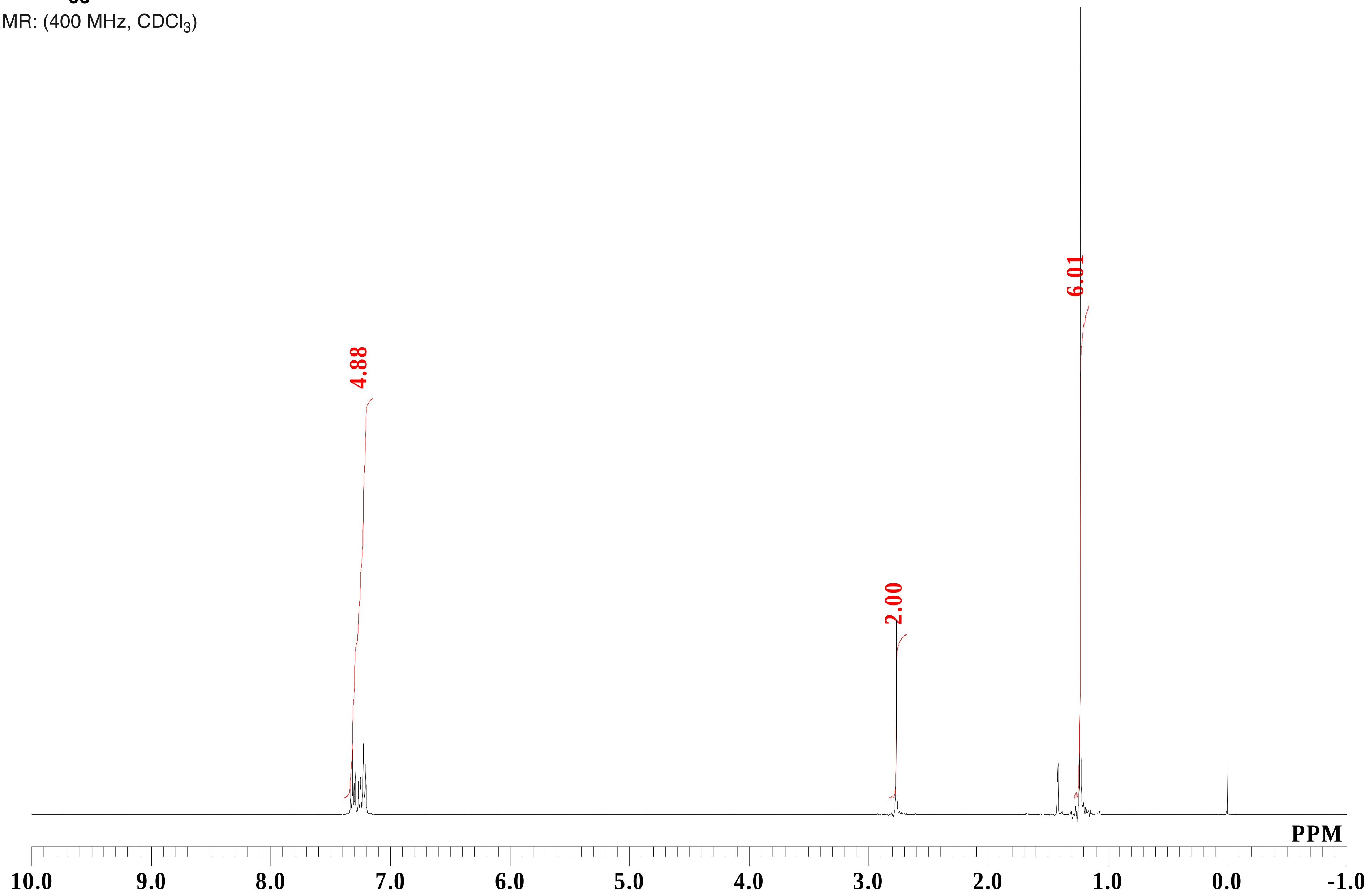

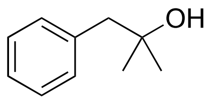

3c

^{13}C NMR: (100 MHz, $CDCl_3$)

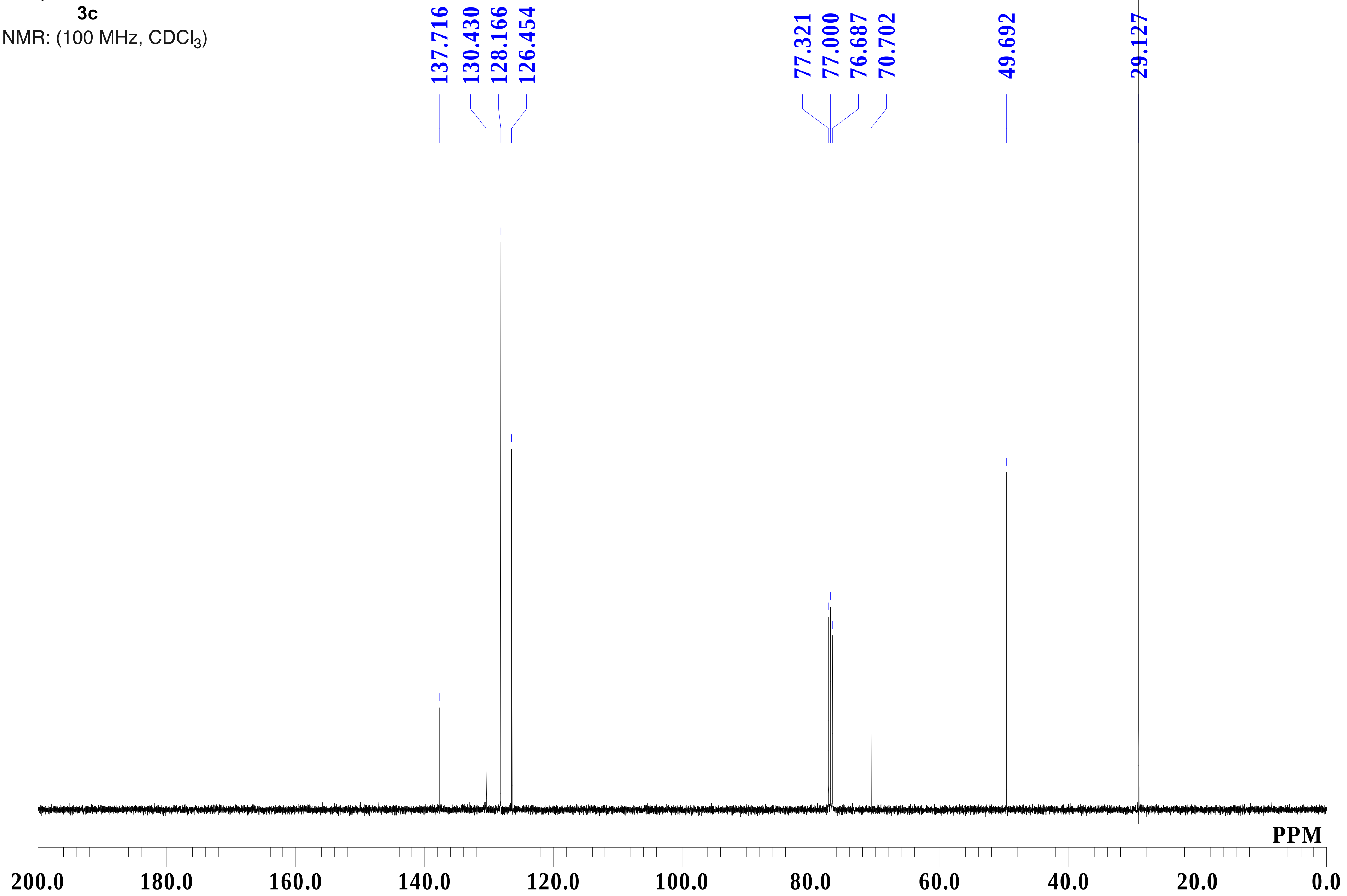

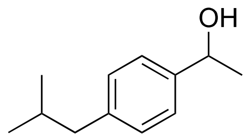

3m

^{1}H NMR: (400 MHz, $CDCl_3$)

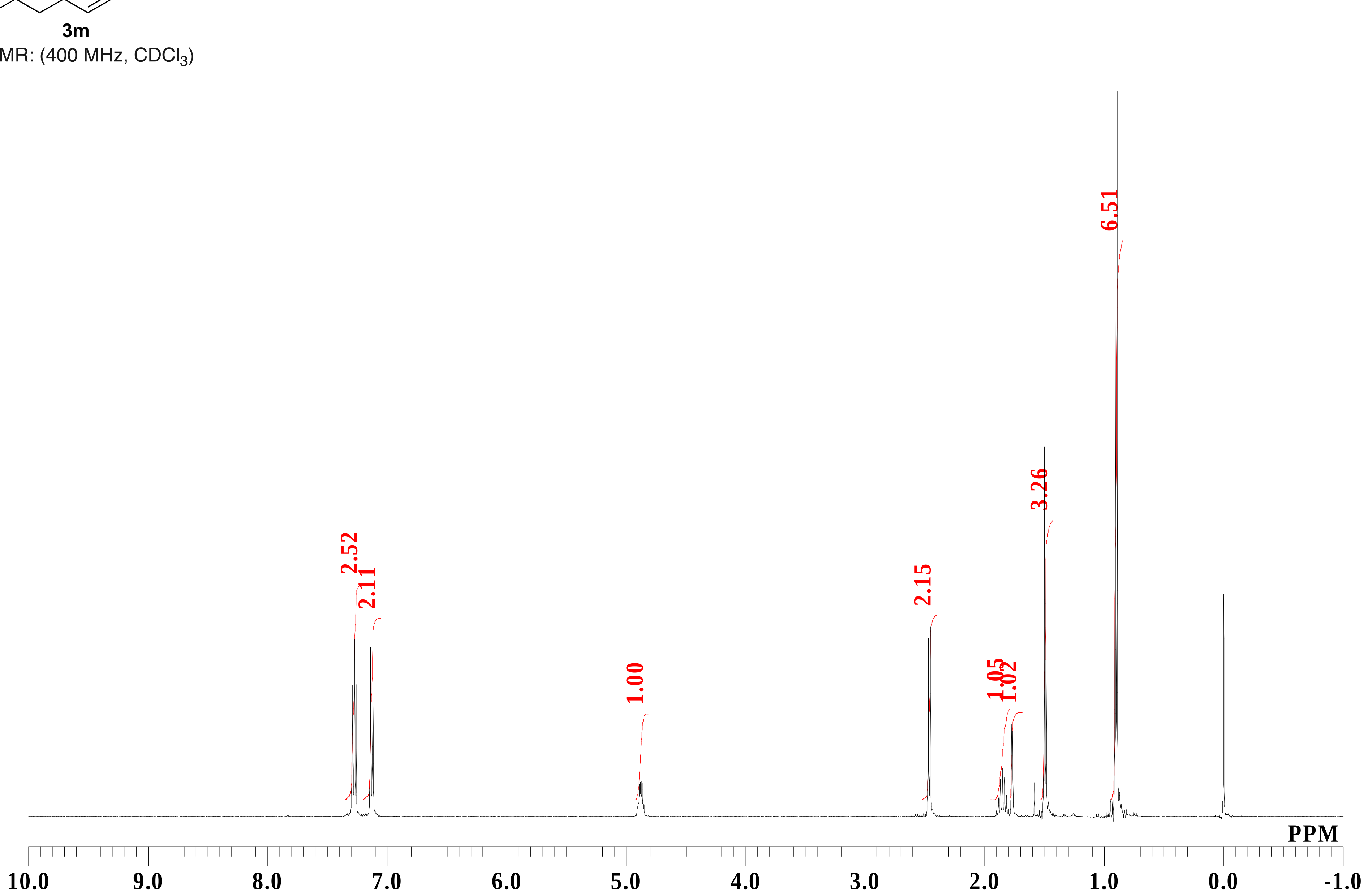

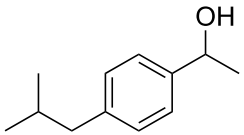

3m

^{13}C NMR: (100 MHz, $CDCl_3$)

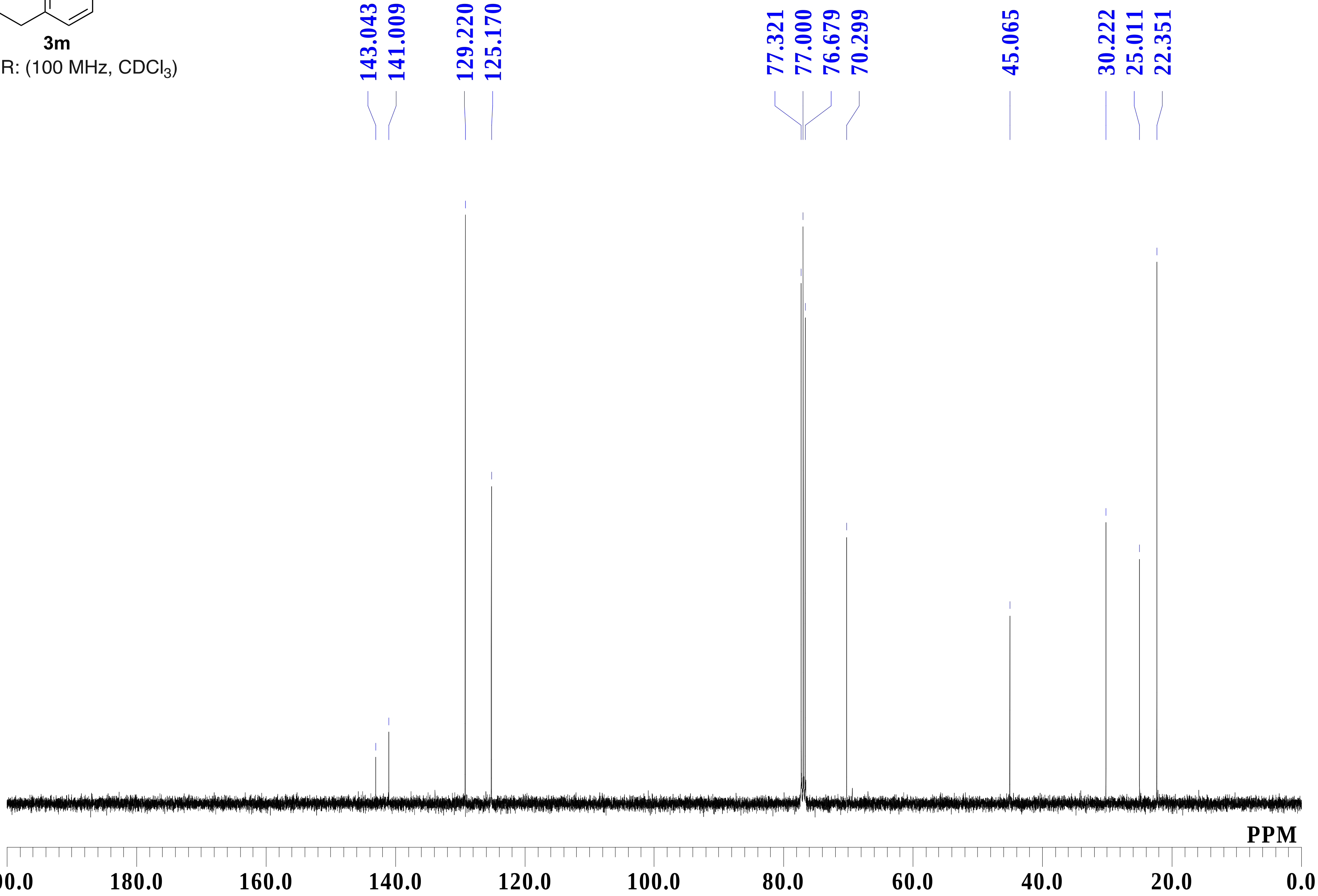

Supplement: File 1 — Experimental procedures, characterization data, copies of the 1H, 13C, and 19F NMR spectra. [file Beilstein_J_Org_Chem-14-1046-s001.pdf]
